# Bidirectional two-sample Mendelian randomization analysis identifies causal associations between migraine and five psychiatric disorders

**DOI:** 10.3389/fneur.2024.1432966

**Published:** 2024-08-05

**Authors:** Wen-Wei Li, Jia-Xin Zhang, Jia Wang, Ya-qing Chen, Sha Lai, Zhi-Kun Qiu

**Affiliations:** Key Specialty of Clinical Pharmacy, The First Affiliated Hospital of Guangdong Pharmaceutical University, Guangzhou, China

**Keywords:** migraine, posttraumatic stress disorder, major depressive disorder, anorexia nervosa, bipolar disorder, schizophrenia, Mendelian randomization

## Abstract

**Background:**

The question of whether a correlation exists between migraine and five psychiatric disorders, including posttraumatic stress disorder (PTSD), major depressive disorder (MDD), anorexia nervosa (AN), bipolar disorder (BIP), and schizophrenia (SCZ), remains a matter of controversy. Hence, this research aims to investigate whether there is a possible association between migraine and five psychiatric disorders.

**Methods:**

We performed a bidirectional 2-sample Mendelian randomization (MR) analysis to assess the causality between migraine and five psychiatric disorders. Genetic associations of PTSD, MDD, AN, BIP, and SCZ were obtained from the Psychiatric Genomics Consortium (PGC) database and genetic associations of migraine with aura and migraine without aura were obtained from the FinnGen dataset. We used the inverse-variance weighted (IVW), weighted median, weighted mode, MR Pleiotropy RESidual Sum and Outlier (MR-PRESSO), and MR Egger regression methods to evaluate the association of genetically predicted exposure with the risk of outcome.

**Results:**

MR demonstrated that MDD was associated with a high risk of migraine without aura (OR = 1.930578, 95% confidence interview (CI): 1.224510, 3.043550, *p* < 0.05), but BIP was related to a low risk of migraine without aura (OR = 0.758650, 95%CI: 0.639601, 0.899858, *p* < 0.05). According to the results of reverse MR, migraine with aura was associated with a high risk of BIP (OR = 1.019100, 95%CI: 1.002538, 1.035935, *p* < 0.05), and migraine without aura was associated with an increased risk of AN (OR = 1.055634, 95%CI: 1.023859, 1.088394, *p* < 0.05).

**Conclusion:**

Our results provide evidence of the potential causal association between migraine and some psychiatric disorders. It may contribute to the prevention of migraine and some psychiatric disorders.

## 1 Introduction

Migraine, mainly divided into migraine with aura and migraine without aura, is a common neurological disorder that has been recognized as the first cause of disability in adult populations under 50 years (1). According to the 2016 Global Burden of Disease study, around 1.04 billion people suffer from migraine worldwide, equivalent to a prevalence of 14.4% overall (2). The typical symptom of migraine is recurrent headaches, usually accompanied by photophobia, nausea, and vomiting (3). It not only severely impacts the quality of life of patients, but also is associated with considerable burden for individuals and the health care system (2, 4).

Psychiatric disorders are another leading contributor to disability worldwide. The study estimated that around 30% of individuals suffer from psychiatric disorders in their lifetime (5) and psychiatric disorders contribute nearly 14% of the global burden of disease (6). According to the research about the genetics of migraine, a shared genetic susceptibility is observed between migraine and several psychiatric disorders, including major depressive disorder (MDD), bipolar disorder (BIP), and schizophrenia (SCZ) (7). Moreover, a large body of research indicated that Migraine patients are comorbid with five major psychiatric disorders including posttraumatic stress disorder (PTSD), anorexia nervosa (AN), MDD, BIP, and SCZ (8–12). It is unclear if any causal relationship exists and directionality between them and potential causal relationships between migraine and five major psychiatric disorders have not been researched directly at a population level to the best of our knowledge. Therefore, investigating the relationship and the directionality between migraine and five major psychiatric disorders could be beneficial for reducing the patient risk of comorbidity of migraine and five major psychiatric disorders and improving the management of migraine or psychiatric disorders.

Most randomized controlled trials (RCT) are time-consuming and expensive. Moreover, there are some unavoidable confounding factors that may affect the outcome of RCTs. Mendelian randomization (MR) is an alternative approach to assess the potential causation and the directionality between migraine and five major psychiatric disorders utilizing relevant genetic variants as instruments (13). Because genetic variants are randomly allocated when the process of gamete formation, the effect of confounding factors could be minimized (14). In this study, the two-sample bidirectional MR was performed to clarify whether the relationship and its direction between migraine (migraine with aura and migraine without aura) and five major psychiatric disorders exist by using summary statistics from non-overlapping samples of previous genome-wide association studies (GWAS) of migraine and five major psychiatric disorders.

## 2 Methods

To assess the causality between migraine (migraine with aura and migraine without aura) and five major psychiatric disorders, the two-sample bidirectional MR was designed. The assumptions of MR design include: (1) the single nucleotide polymorphisms (SNPs) were used as instrumental variables (IVs) for exposure; (2) IVs are not related to the confounders; (3) IVs affect the risk of outcome only by the exposure. Psychiatric disorders were first used as a risk factor and migraine as an outcome, then the reverse, from two non-overlapping datasets. This research was based on the publicly available dataset and the approval of every research can be discovered in the original studies.

### 2.1 Genetic associated with psychiatric disorders

Genetic associations with five major psychiatric disorders were obtained from the publicly available GWAS among individuals of European ancestry contributed from the Psychiatric Genomics Consortium (PGC) database. The respective sample sizes were as follows: AN (16,992 cases and 55,525 controls), BIP (41,917 cases and 371,549 controls), PTSD (23,212 cases and 151,447 controls), MDD (246,363 cases and 561,190 controls), and SCZ (53,386 cases and 77,258 controls).

The SNPs of exposure, selected as IVs, usually achieved genome-wide significant threshold (*p* < 5 × 10^−8^) and the mean F statistic (F). In this study, the significant threshold was used to select SNPs of SCZ, MDD and BIP. Due to insufficient numbers of SNP for the other four psychiatric disorders, an arbitrary threshold of 5 × 10^−6^ and *F* > 10 was used to select SNPs for the sufficient number of IVs for AN and PTSD.

### 2.2 Genetic associated with migraine

The SNPs of migraine among individuals of European ancestry, migraine with aura and migraine without aura, were extracted from the FinnGen dataset. The respective sample sizes were as follows: migraine with aura (3,541 cases and 176,107 controls) and migraine without aura (3,215 cases and 176,107 controls).

Based on the genome-wide significant threshold, there were less numbers of SNPs. Therefore, an arbitrary threshold of 1 × 10^−4^ and *F* > 10 was used to select SNPs for the sufficient number of IVs of migraine with aura and migraine without aura. The GWAS IDs of migraine with aura and migraine without aura were finn-b-G6 MIGRAINE WITH AURA and finn-b-G6 MIGRAINE NO AURA.

### 2.3 Statistical analysis

To remove linkage disequilibrium (LD), the IVs (r^2^ > 0.01 and distance <10,000 kb) associated with predicted exposures were removed. The SNPs associated with the exposure were extracted from the outcome, if SNPs for the exposure were not extracted, they were further removed. And then, to prevent the influence of confounding factors, the SNPs that have been found to be associated with secondary traits were removed by searching the GWAS Catalog.^1^ The SNPs included in the final MR analysis were displayed in Supplementary Table S1.

The inverse-variance weighted (IVW), weighted median, weighted mode, and MR Egger regression methods were used to evaluate the association of genetically predicted exposure with the risk of outcome (15). Among them, the IVW was chosen as the main method which calculated the Wald estimates via effect estimates of each IV of exposure and risk of outcome. Weighted median, weighted mode, and MR Egger regression methods were performed to assess the robustness of the association and the presence of directional pleiotropy was identified by using MR-Egger regression (16–19). In addition, the significant outliers will be detected and removed to correct the horizontal pleiotropic effect via MR Pleiotropy RESidual Sum and Outlier (MR PRESSO) test. The Cochran’s Q test was used to examine the heterogeneity of all SNPs from different datasets and the scatter plots were generated (20). Leave-one-out analysis was conducted to assess the potential influence of a particular variant on the estimates by performing an IVW method on remaining SNPs after excluding each SNP. The associations between genetically predicted exposure and risk of outcome were expressed as odds ratios (ORs) with their 95% confidence intervals (CIs).

All statistical analyses were performed with the “TwoSampleMR” and “MR-PRESSO” packages in R software 4.2.1 (21).

## 3 Results

### 3.1 Genetically predicted psychiatric disorders on migraine

After removing LD and screening *p*-value, 55, 54, 50, 22, and 155 SNPs were obtained for AN, BIP, MDD, PTSD, and SCZ, respectively. To avoid the influence of confounding factors, 13, 25, 28, 7, and 60 SNPs were removed for AN, BIP, MDD, PTSD, and SCZ after searching the GWAS Catalog, respectively. All the F of SNPs were above 10, which suggested weak instrument bias was unlikely. Detailed information about the selected SNPs of five mental diseases and two migraine-related phenotypes was listed in Supplementary Table S1.

For BIP as exposure, the IVW result suggested that genetically predicted BIP (OR = 0.758650, 95%CI: 0.639601, 0.899858, *p* < 0.05) was significantly associated with the risk of migraine without aura. The results of the weighted median were consistent, but no significant association was identified in the MR-Egger model.

**Figure 1:**
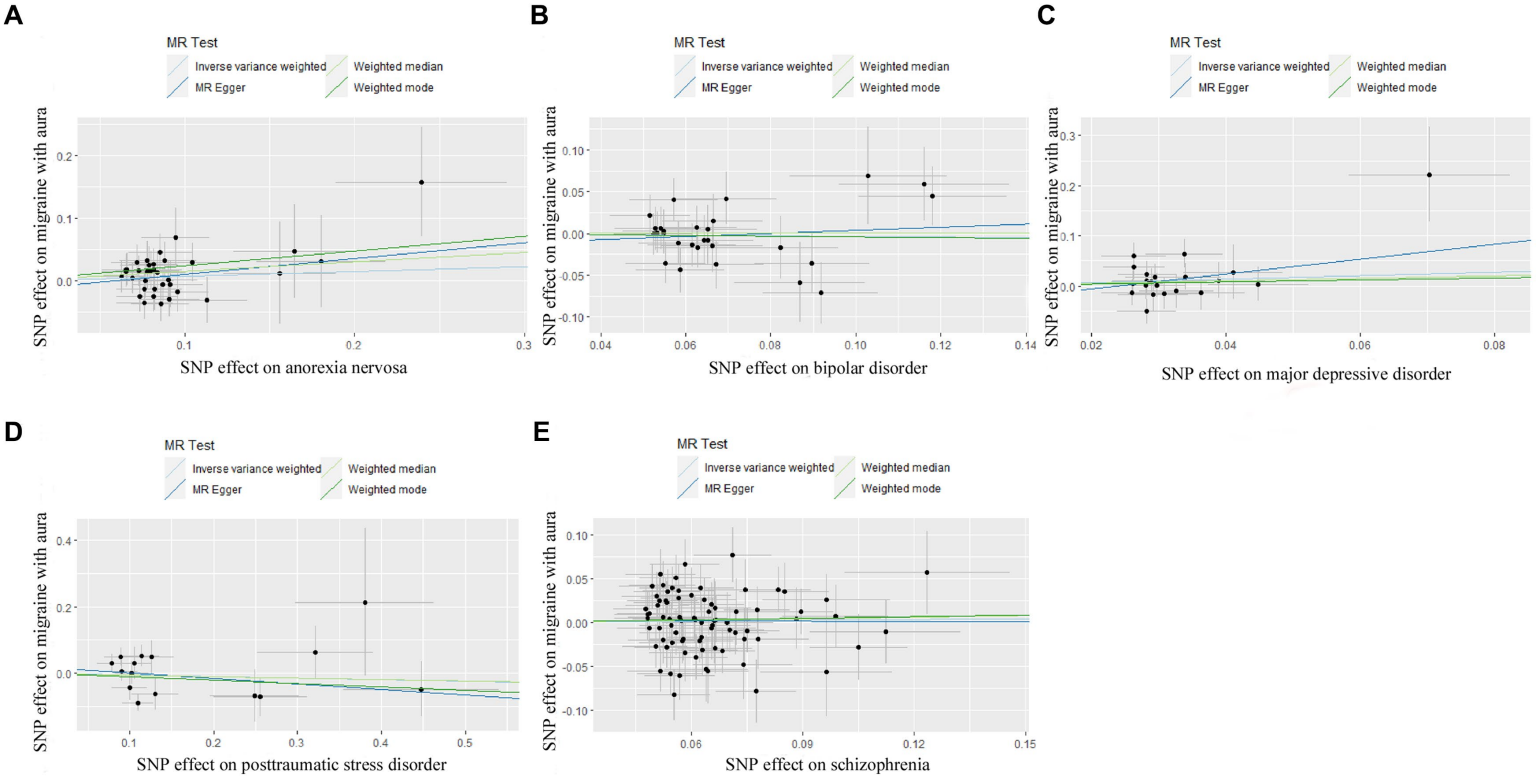
Scatter plot of genetic correlation for psychiatric disorders on migraine with aura by different MR analysis methods. **(A)** The causal effect of anorexia nervosa on migraine with aura; **(B)** The causal effect of bipolar disorder on migraine with aura; **(C)** The causal effect of major depressive disorder on migraine with aura; **(D)** The causal effect of posttraumatic stress disorder on migraine with aura; **(E)** The causal effect of schizophrenia on migraine with aura.

**Figure 2:**
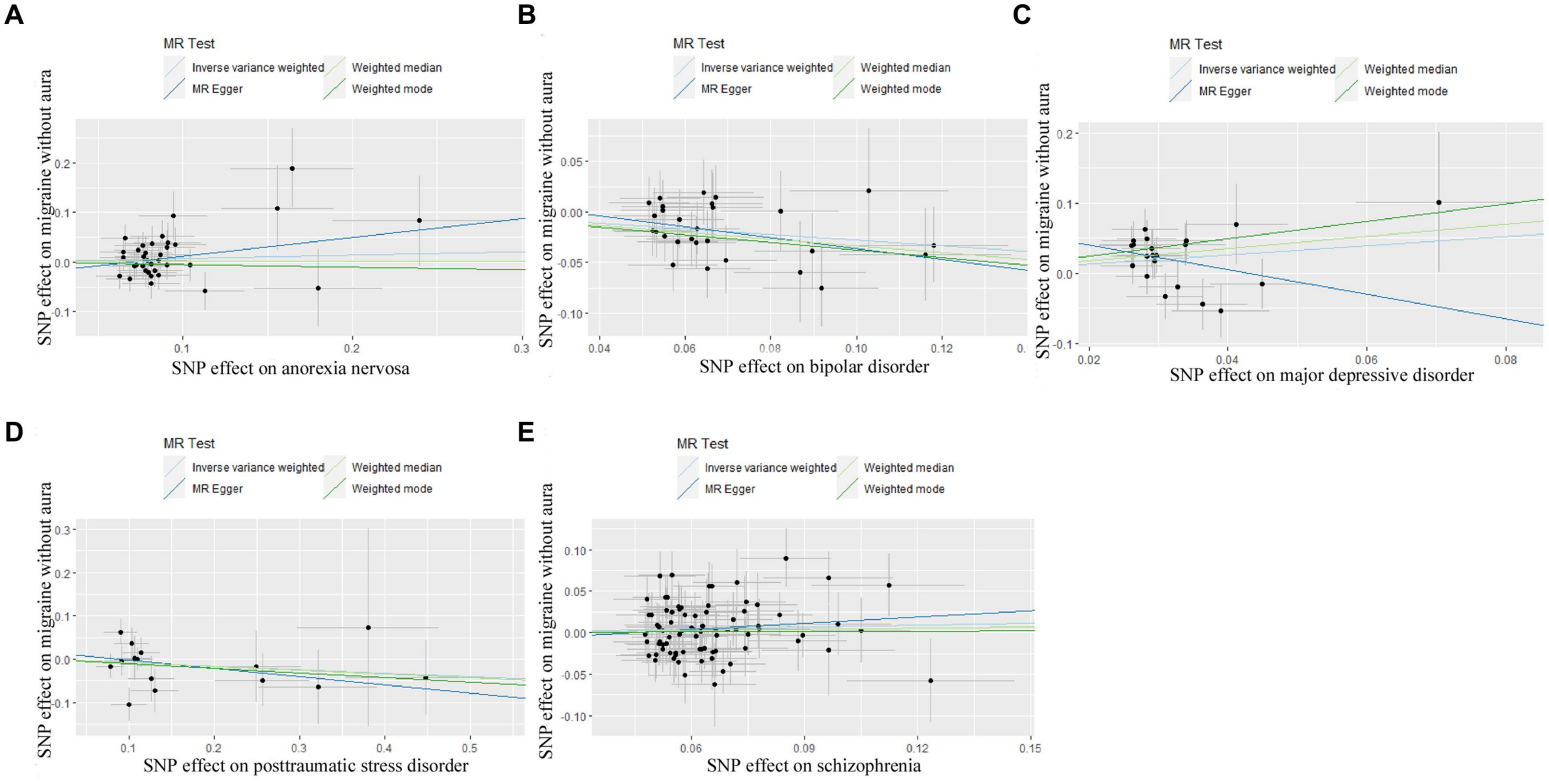
Scatter plot of genetic correlation for psychiatric disorders on migraine without aura by different MR analysis methods. **(A)** The causal effect of anorexia nervosa on migraine without aura; **(B)** The causal effect of bipolar disorder on migraine without aura; **(C)** The causal effect of major depressive disorder on migraine without aura; **(D)** The causal effect of posttraumatic stress disorder on migraine without aura; **(E)** The causal effect of schizophrenia on migraine without aura.

In the case of MDD, the IVW result suggested that genetically predicted MDD (OR = 1.930578, 95%CI: 1.224510, 3.043550, *p* < 0.05) was related to the risk of migraine without aura. Similarly, the results of the weighted median were consistent, but no significant association was identified in the MR-Egger model. No significant association was identified in the other three psychiatric disorders (Figures 1, 2). Detailed information about Mendelian randomization was listed in Table 1.

**Table 1:** Results of MR analysis for psychiatric disorders on migraine.

Exposure	Outcome	Method	OR	LCI, UCI	*p*-value
AN	Migraine with aura	IVW	1.078370	(0.957002, 1.215130)	2.16E − 01
		MR-Egger	1.284573	(0.724130, 2.278771)	3.98E − 01
		Weighted median	1.161343	(0.981670, 1.373901)	8.60E − 02
		Weighted mode	1.266217	(0.860874, 1.862416)	2.39E − 01
		MR-PRESSO (Outlier-corrected)			NA
	Migraine without aura	IVW	1.071361	(0.942887, 1.217342)	2.90E − 01
		MR-Egger	1.458162	(0.790049, 2.691273)	0.236
		Weighted median	1.005225	(0.839080, 1.204268)	9.55E − 01
		Weighted mode	0.946516	(0.658125. 1.361282)	7.69E − 01
		MR-PRESSO (Outlier-corrected)			NA
BIP	Migraine with aura	IVW	0.967731	(0.822986, 1.137934)	0.692
		MR-Egger	1.199052	(0.584442, 2.460002)	0.625
		Weighted median	1.007684	(0.799529, 1.270033)	0.948
		Weighted mode	0.958561	(0.597444, 1.537950)	0.862
		MR-PRESSO (Outlier-corrected)			NA
	Migraine without aura	IVW	0.758650	(0.639601, 0.899858)	**0.002**
		MR-Egger	0.584553	(0.274084, 1.246705)	0.176
		Weighted median	0.716039	(0.566756, 0.904643)	**0.005**
		Weighted mode	0.685708	(0.443214, 1.060877)	0.102
		MR-PRESSO (Outlier-corrected)			NA
MDD	Migraine with aura	IVW	1.412851	(0.920176, 2.169310)	1.14E − 01
		MR-Egger	4.407621	(0.332731, 58.386793)	2.74E − 01
		Weighted median	1.279335	(0.719938, 2.273386)	4.01E − 01
		Weighted mode	1.225612	(0.719938, 2.879962)	6.46E − 01
		MR-PRESSO (Outlier-corrected)			NA
	Migraine without aura	IVW	1.930578	(1.224510, 3.043550)	**5.00E** − **03**
		MR-Egger	0.173124	(0.013255, 2.261218)	1.97E − 01
		Weighted median	2.385532	(1.317120, 4.320353)	**4.00E** − **03**
		Weighted mode	3.469590	(1.040922, 11.56480)	5.60E − 02
		MR-PRESSO (Outlier-corrected)			NA
PTSD	Migraine with aura	IVW	0.955002	(0.775192, 1.175611)	0.664
		MR-Egger	0.848160	(0.537310, 1.338845)	0.492
		Weighted median	0.957320	(0.758093, 1.208903)	0.714
		Weighted mode	0.903670	(0.638486, 1.278992)	0.577
		MR-PRESSO (Outlier-corrected)			6.21E − 01
	Migraine without aura	IVW	0.923013	(0.777052, 1.096392)	0.362
		MR-Egger	0.828023	(0.568303, 1.206438)	0.344
		Weighted median	0.916555	(0.734721, 1.143390)	0.440
		Weighted mode	0.900008	(0.679789, 1.191568)	0.474
		MR-PRESSO (Outlier-corrected)			NA
SCZ	Migraine with aura	IVW	1.032328	(0.925982, 1.150887)	5.66E − 01
		MR-Egger	0.994695	(0.583694, 1.695096)	9.84E − 01
		Weighted median	1.059464	(0.923418, 1.215554)	4.10E − 01
		Weighted mode	1.061196	(0.783783, 1.436796)	7.02E − 01
		MR-PRESSO (Outlier-corrected)			NA	
	Migraine without aura	IVW	1.078597	(0.973281, 1.195308)	1.49E − 01
		MR-Egger	1.287354	(0.779007, 2.127427)	3.27E − 01
		Weighted median	1.047003	(0.904512, 1.211942)	5.38E − 01
		Weighted mode	1.012760	(0.724192, 1.416315)	9.41E − 01
		MR-PRESSO (Outlier-corrected)			NA

LCI, Low confidence intervals; UCI, UP confidence intervals; IVW, Inverse variance weighted; MR-PRESSO, Mendelian Randomisation Pleiotropy RESidual Sum and Outlier; *P*, *P*-value; The bold and underline indicate *P*-value < 0.05.

**Figure 3:**
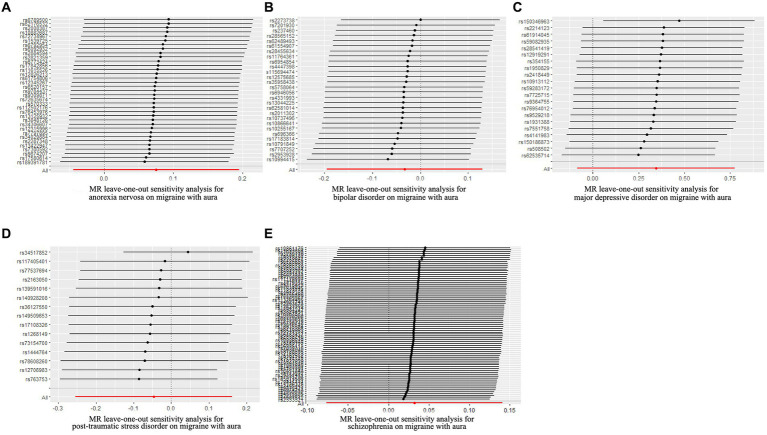
Leave-one-out sensitivity analysis for psychiatric disorders on migraine with aura. **(A)** The sensitivity analysis for anorexia nervosa on migraine with aura; **(B)** The sensitivity analysis for bipolar disorder on migraine with aura; **(C)** The sensitivity analysis for major depressive disorder on migraine with aura; **(D)** The sensitivity analysis for posttraumatic stress disorder on migraine with aura; **(E)** The sensitivity analysis for schizophrenia on migraine with aura.

**Figure 4:**
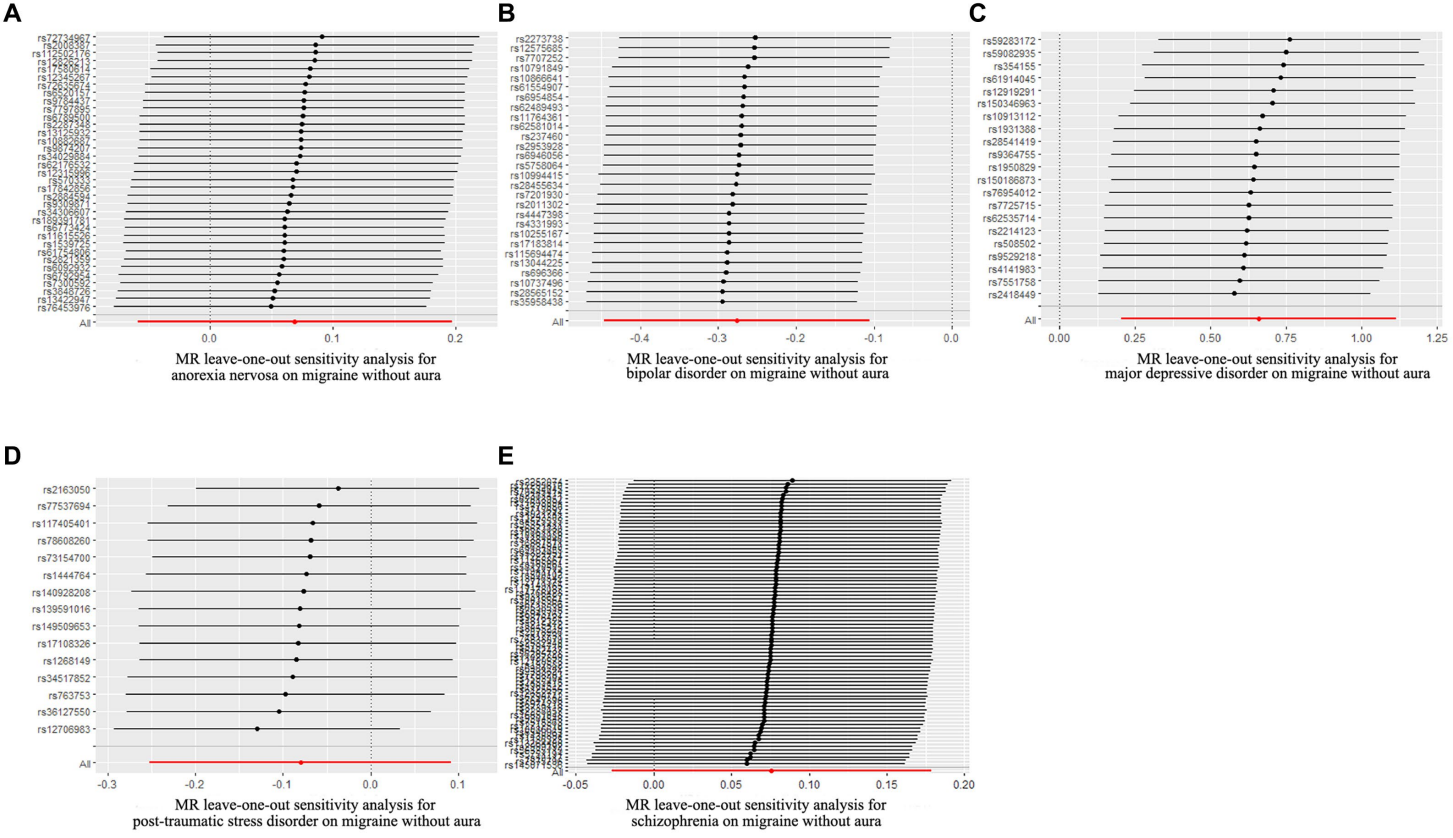
Leave-one-out sensitivity analysis for psychiatric disorders on migraine without aura. **(A)** The sensitivity analysis for anorexia nervosa on migraine without aura; **(B)** The sensitivity analysis for bipolar disorder on migraine without aura; **(C)** The sensitivity analysis for major depressive disorder on migraine without aura; **(D)** The sensitivity analysis for posttraumatic stress disorder on migraine without aura; **(E)** The sensitivity analysis for schizophrenia on migraine without aura.

The pleiotropic effect of SCZ on migraine with aura was detected by global test. No pleiotropic effect was detected in other MR analyses (Table 2). The results of Cochran’s Q test indicated that possible heterogeneity was detected in MR analysis of PTSD on migraine with aura. For the visualization of heterogeneity, funnel plots were shown in Supplementary Figures S1, S2. The MR PRESSO test demonstrated the effects were consistent with IVW after detecting and removing significant outliers. The results of the Leave-one-out analysis (Figures 3, 4) showed that significant associations were still observed in all IVW analyses after the exclusion of each SNP and forest plots of MR analysis were shown in Supplementary Figures S3, S4.

**Table 2:** Results of MR Steiger direction test for psychiatric disorders on migraine.

Exposure	Outcome	IVW Q statistic(*P*^a^)	MR-Egger intercept(*P*^b^)	MR-PRESSO	
				Global test *P*^b^	Outlier-corrected *P*
AN	Migraine with aura	0.902	0.545	0.888	NA
BIP		0.625	0.554	0.633	NA
MDD		0.295	0.392	0.293	NA
PTSD		0.021	0.574	0.196	0.621
SZC		0.051	0.889	0.048	NA
AN	Migraine without aura	0.418	0.321	0.444	NA
BIP		0.965	0.495	0.971	NA
MDD		0.277	0.078	0.296	NA
PTSD		0.282	0.533	0.31	NA
SZC		0.414	0.483	0.42	NA

IIVW, inverse variance weighted; MR, Mendelian randomization; MR-PRESSO, MR-pleiotropy residual sum and outlier. a, Cochrane’s Q test with *p*-value<0.05 indicates a possible heterogeneity; b, MR-Egger test with *p*-value<0.05 indicates horizontal pleiotropy.

### 3.2 Genetically predicted migraine on psychiatric disorders

**Figure 5:**
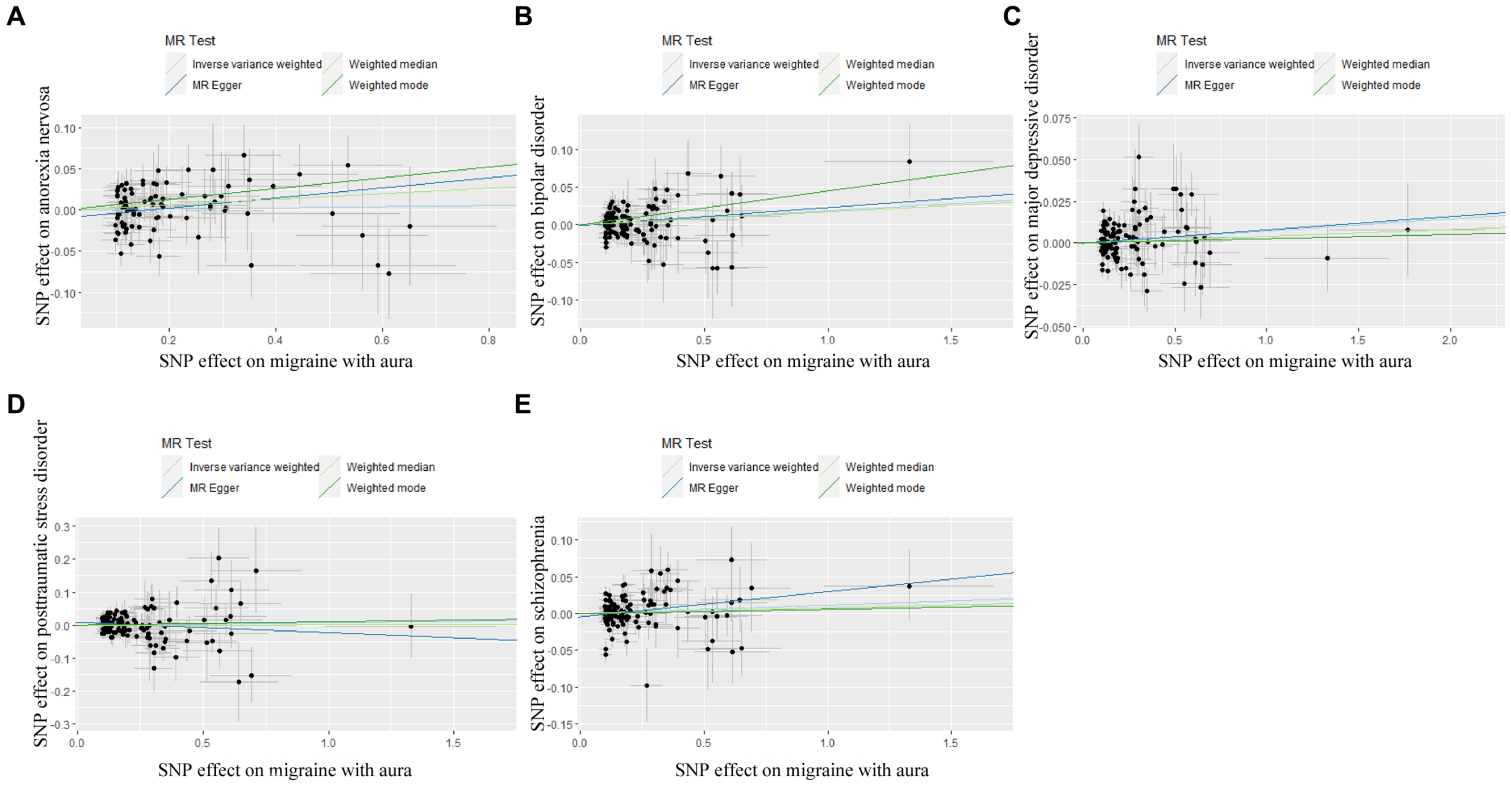
Scatter plot of genetic correlation for migraine with aura on psychiatric disorders by different MR analysis methods. **(A)** The causal effect of migraine with aura on anorexia nervosa; **(B)** The causal effect of migraine with aura on bipolar disorder; **(C)** The causal effect of migraine with aura on major depressive disorder; **(D)** The causal effect of migraine with aura on posttraumatic stress disorder; **(E)** The causal effect of migraine with aura on schizophrenia.

**Figure 6:**
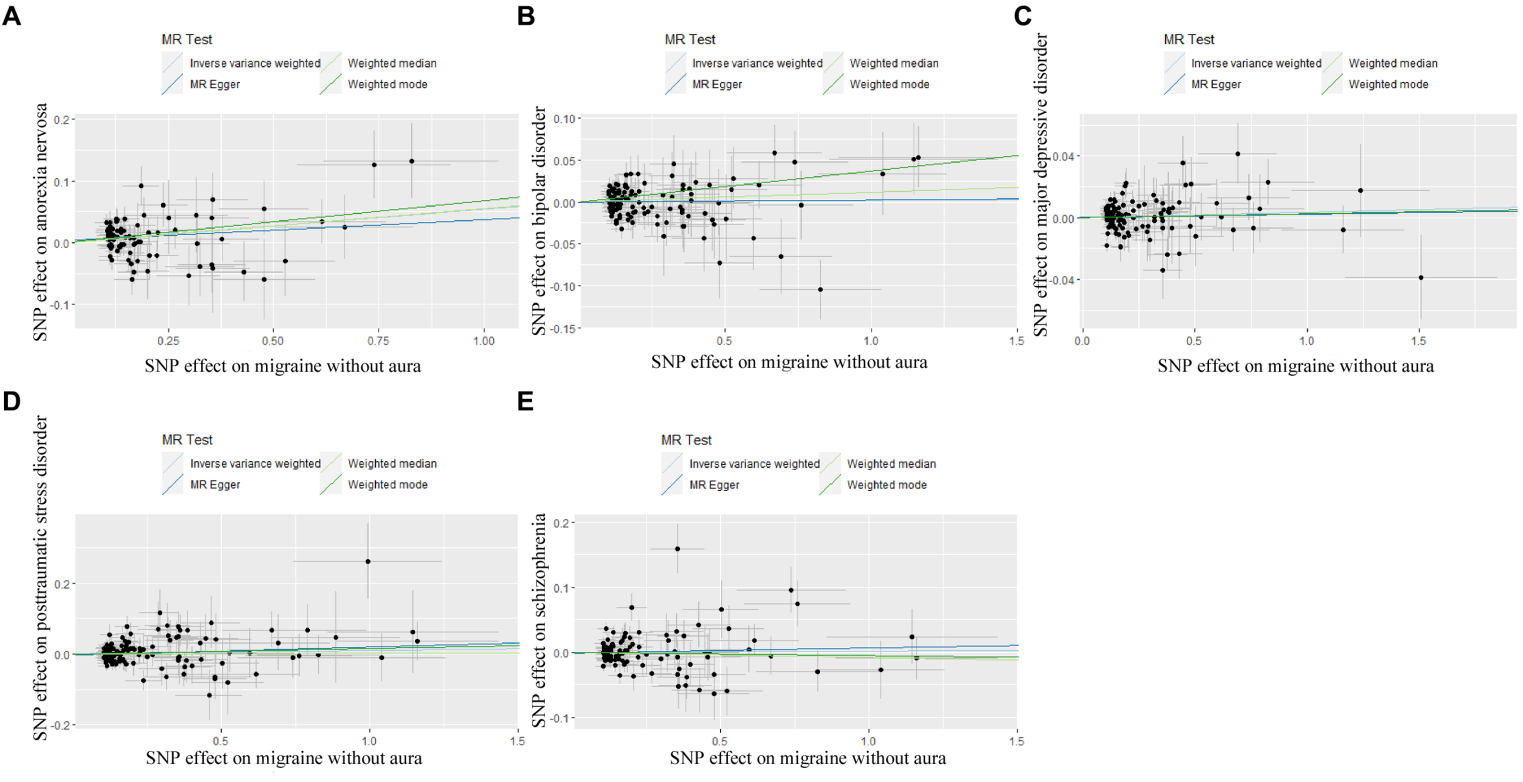
Scatter plot of genetic correlation for migraine without aura on psychiatric disorders by different MR analysis methods. **(A)** The causal effect of migraine without aura on anorexia nervosa; **(B)** The causal effect of migraine without aura on bipolar disorder; **(C)** The causal effect of migraine without aura on major depressive disorder; **(D)** The causal effect of migraine without aura on posttraumatic stress disorder; **(E)** The causal effect of migraine without aura on schizophrenia.

In reverse Mendelian randomization (Figures 5, 6), 134 SNPs were selected from migraine with aura and 137 SNPs were selected from migraine without aura. All the F of SNPs were above 10, which suggested weak instrument bias was unlikely. The IVW results indicated that migraine with aura was significantly associated with BIP (OR = 1.019100, 95%CI: 1.002538, 1.035935, *p* < 0.05) and migraine without aura was related to AN (OR = 1.055634, 95%CI: 1.023859, 1.088394, *p* < 0.05). Moreover, the weighted median results of migraine without aura on AN were in line with the IVW results. Detailed information about reverse Mendelian randomization was listed in Table 3.

**Table 3:** Results of reverse MR analysis for migraine on psychiatric disorders.

Exposure	Outcome	Method	OR	LCI, UCI	*p*-value
Migraine with aura	AN	IVW	1.006727	(0.974680,1.039827)	0.685
		MR Egger	1.062857	(0.985118,1.146731)	0.119
		Weighted median	1.033826	(0.992320,1.077068)	0.112
		Weighted mode	1.066992	(0.972153,1.171084)	0.176
		MR-PRESSO(Outlier-corrected)			NA
	BIP	IVW	1.019100	(1.002538,1.035935)	**0.0236**
		MR Egger	1.024008	(0.989583,1.059631)	0.177
		Weighted median	1.017297	(0.992820,1.042377)	0.168
		Weighted mode	1.046105	(0.996254,1.098450)	0.073
		MR-PRESSO(Outlier-corrected)			NA
	MDD	IVW	1.007280	(0.998681,1.015953)	0.097
		MR Egger	1.007962	(0.991794,1.024390)	0.338
		Weighted median	1.004003	(0.993368,1.014753)	0.462
		Weighted mode	1.002495	(0.983389,1.021972)	0.800
		MR-PRESSO(Outlier-corrected)			0.074
	PTSD	IVW	1.007606	(0.982590,1.033259)	0.555
		MR Egger	0.969507	(0.920408,1.021225)	0.245
		Weighted median	1.001709	(0.963798,1.041111)	0.931
		Weighted mode	1.010268	(0.936959,1.089313)	0.791
		MR-PRESSO(Outlier-corrected)			NA
	SCZ	IVW	1.011489	(0.991015,1.032387)	0.274
		MR Egger	1.034523	(0.991540,1.079369)	0.120
		Weighted median	1.007680	(0.984490,1.031417)	0.520
		Weighted mode	1.005954	(0.965647,1.047941)	0.777
		MR-PRESSO(Outlier-corrected)			**0.028**
Migraine without aura	AN	IVW	1.055634	(1.023859,1.088394)	**0.001**
		MR Egger	1.034408	(0.965840,1.107844)	0.337
		Weighted median	1.057501	(1.014431,1.102399)	**0.008**
		Weighted mode	1.070177	(0.986179,1.161329)	0.108
		MR-PRESSO(Outlier-corrected)			NA
	BIP	IVW	1.001901	(0.987118,1.016905)	0.802
		MR Egger	1.002351	(0.975220,1.030237)	0.867
		Weighted median	1.011464	(0.988974,1.034465)	0.320
		Weighted mode	1.037596	(0.995942,1.080992)	0.080
		MR-PRESSO(Outlier-corrected)			NA
	MDD	IVW	1.003428	(0.996163,1.010747)	0.356
		MR Egger	1.001962	(0.988760,1.015341)	0.773
		Weighted median	1.002491	(0.992756,1.012323)	0.617
		Weighted mode	1.002511	(0.983973,1.021399)	0.792
		MR-PRESSO(Outlier-corrected)			0.196
	PTSD	IVW	1.009296	(0.987208,1.031877)	0.412
		MR Egger	1.022957	(0.982261,1,065338)	0.275
		Weighted median	1.000536	(0.965516,1.036825)	0.976
		Weighted mode	1.016030	(0.952126,1.084222)	0.632
		MR-PRESSO(Outlier-corrected)			NA	
	SCZ	IVW	1.001867	(0.984018,1.020041)	0.839
		MR Egger	1.007663	(0.975289,1.041113)	0.648
		Weighted median	0.992003	(0.969509,1.015019)	0.493
		Weighted mode	0.994816	(0.959604,1.031320)	0.778
		MR-PRESSO(Outlier-corrected)			0.754

LCI, Low confidence intervals; UCI, UP confidence intervals; IVW, Inverse variance weighted; MR-PRESSO, Mendelian Randomisation Pleiotropy RESidual Sum and Outlier; *P*, *P*-value; The bold and underline indicate *P*-value < 0.05.

**Figure 7:**
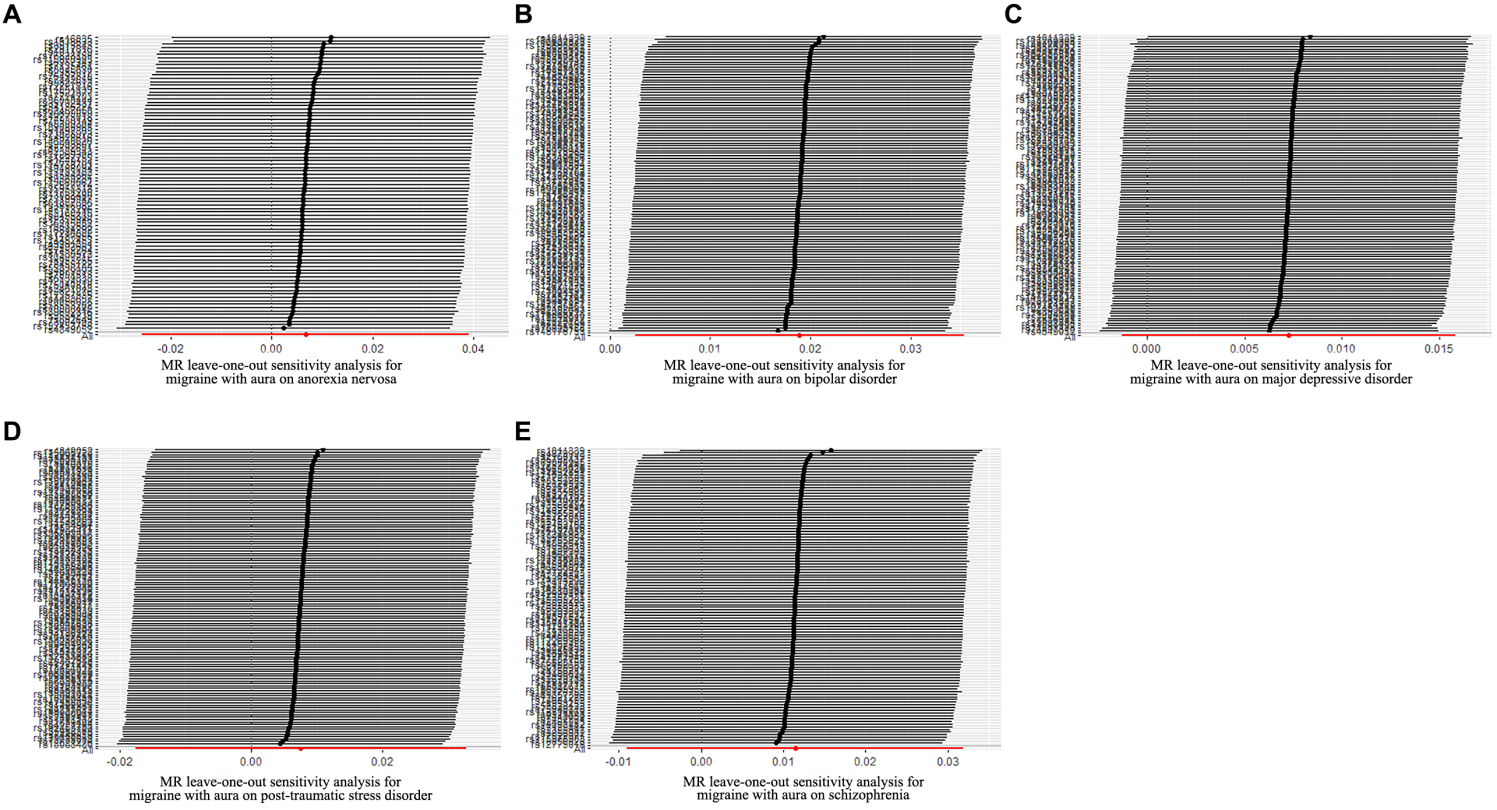
Leave-one-out sensitivity analysis for migraine with aura on psychiatric disorders. **(A)** The sensitivity analysis for migraine with aura on anorexia nervosa; **(B)** The sensitivity analysis for migraine with aura on bipolar disorder; **(C)** The sensitivity analysis for migraine with aura on major depressive disorder; **(D)** The sensitivity analysis for migraine with aura on posttraumatic stress disorder; **(E)** The sensitivity analysis for migraine with aura on schizophrenia.

**Figure 8:**
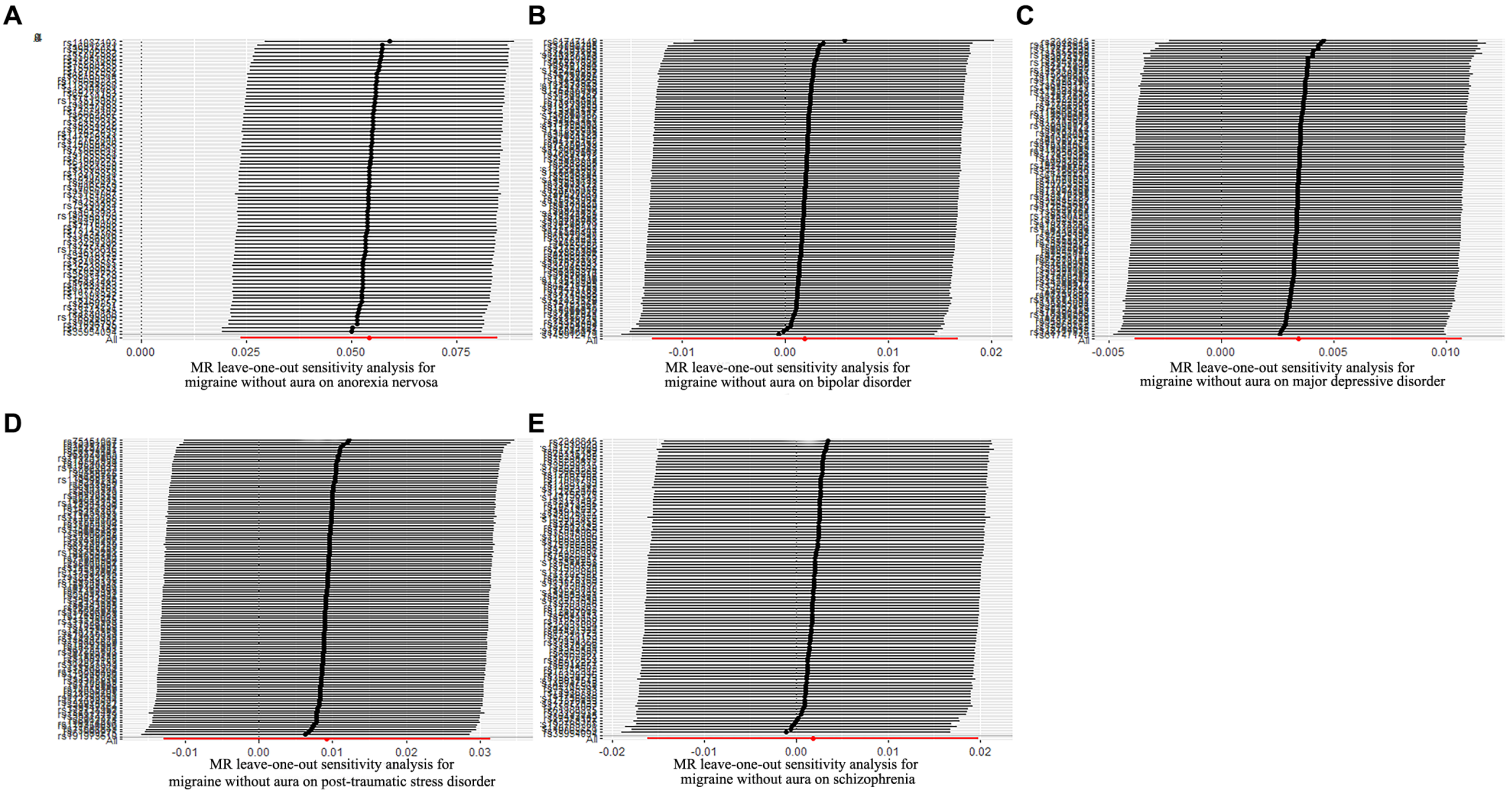
Leave-one-out sensitivity analysis for migraine without aura on psychiatric disorders. **(A)** The sensitivity analysis for migraine without aura on anorexia nervosa; **(B)** The sensitivity analysis for migraine without aura on bipolar disorder; **(C)** The sensitivity analysis for migraine without aura on major depressive disorder; **(D)** The sensitivity analysis for migraine without aura on posttraumatic stress disorder; **(E)** The sensitivity analysis for migraine without aura on schizophrenia.

The possible heterogeneity was observed in MR analysis of migraine with aura on AN, MDD, and SCZ, as well as migraine without aura in relation to the same conditions. The global test identified the pleiotropic effect of migraine with aura on AN, MDD, and SCZ, and migraine without aura on MDD and SCZ. For the visualization of heterogeneity of reverse MR, funnel plots were shown in Supplementary Figures S5, S6. The MR PRESSO test showed that migraine with aura was significantly associated with SCZ after detecting and removing significant outliers, but other effects were consistent with IVW (Table 4). The results of the Leave-one-out analysis (Figures 7, 8) showed that significant associations were still observed in all IVW analyses after the exclusion of each SNP and forest plots of reverse MR analysis were shown in Supplementary Figures S7, S8.

**Table 4:** Results of MR Steiger direction test for migraine on psychiatric disorders.

Exposure	Outcome	Univariable MR			
		IVW Q statistic(*P*^a^)	MR-Egger intercept(*P*^b^)	MR-PRESSO	
				Global test *P*^b^	Outlier-corrected *P*
Migraine with aura	ADHD	5.60E − 01	0.425	0.565	NA
	AN	0.005	0.126	0.005	NA
	BIP	0.12	0.754	0.217	NA
	MDD	4.63E − 06	0.923	3.33E − 04	0.074
	OCD	0.439	0.905	0.436	NA
	PTSD	0.342	0.100	0.34	NA
	SZC	1.97E − 08	0.238	3.33E − 04	0.028
Migraine without aura	ADHD	0.393	0.357	0.412	NA
	AN	0.047	0.518	0.058	NA
	BIP	0.267	0.97	0.263	NA
	MDD	0.022	0.796	0.021	0.196
	OCD	0.483	0.013	0.484	NA
	PTSD	0.606	0.44	0.62	NA
	SZC	5.52E − 05	0.679	3.33E − 04	7.54E − 01

IVW, inverse variance weighted; MR, Mendelian randomization; MR-PRESSO, MR-pleiotropy residual sum and outlier. a, Cochrane’s Q test with *p*-value < 0.05 indicates a possible heterogeneity; b, MR-Egger test with p-value<0.05 indicates horizontal pleiotropy.

## 4 Discussion

In the present study, bidirectional MR analysis was used to determine the association between five major psychiatric disorders and migraine (migraine without aura and migraine with aura). The evidence indicated that genetically predicted MDD was associated with a high risk of migraine without aura, but BIP was related to a low risk of migraine without aura. According to the results of reverse MR, migraine with aura was associated with a high risk of BIP, and migraine without aura was associated with an increased risk of AN.

Growing evidence from the research indicated that there is a high comorbidity rate between BIP and migraine (22, 23). Unfortunately, migraine was not divided into two different subtypes for the study’s investigation in those research. There are numerous risk factors that overlap between BIP and migraine, such as oxidative stress and disruptions in inflammatory cytokines, especially related to pro-inflammatory and anti-inflammatory factors. Serotonin (5-HT), considered to be associated with migraine and BIP, is involved in behavioral functions including locomotor activity, pain sensitivity, and emotion (24). An imbalance of 5-HT neurotransmitters could potentially lead to various behavioral dysregulations. Research has found that the central 5-HT activity in patients with BIP is reducer than healthy individuals and low levels of 5-HT in the brain are also detected between attacks in migraine patients (25, 26). The function of glutamate is crucial in regulating neuronal excitability, with significant implications for neuroprotection and neuroplasticity in emotional disorders (27). Research has also suggested that glutamate is involved in pain transmission, central sensitization, and cortical diffusion inhibition processes (28, 29). However, Significant upregulation of glutamate levels was observed in the anterior cingulate cortex of patients with BIP and increased concentrations of glutamate have been found in the brain and peripheral circulation of individuals with migraine, particularly during migraine attacks (30). It indicated that the disorder of glutamate system is related to BIP and migraine. Additionally, abnormalities in the dopamine pathway are closely intertwined with these conditions (24). Studies have found that individuals with migraine with aura exhibit higher levels of glutamate intake in their bodies compared to normal individuals, whereas those with migraine without aura demonstrate lower levels of glutamate intake compared to normal individuals (31). If parents experience migraines, there is an increased likelihood of their offspring developing bipolar disorder, even if the parents do not have bipolar disorder themselves (32, 33). It is consistent with our MR results that migraine with aura was associated with a high risk of BIP. However, there is currently no research supporting our other conclusion that BIP was related to a low risk of migraine without aura.

A large Polish migraine cohort study and a study of registry for Migraine found that the proportion of migraine patients with MDD was 20.3% and 10.8%, respectively (34, 35). Moreover, most previous studies indicated a relationship between migraine and MDD (8, 36), but our research only found evidence that MDD was associated with a high risk of migraine without aura. In a study of 272 MDD patients, 26.1% were migraine without aura patients, while only 6.6% were migraine with aura (37). It provided some support for our research results. Previous research has observed a decrease in the dopamine system, an imbalance of the 5-HT neurotransmitters, and genetic polymorphism of the methylenetetrahydrofolate reductase enzyme in individuals with migraine or MDD compared to normal individuals (38, 39). Decreased levels of 5-HT have been identified as significant biomarkers in individuals with MDD, and the clinical symptoms of MDD can be relieved by using 5-HT reuptake inhibitors (40). Moreover, some studies suggested that migraine is possibly induced by low levels of 5-HT, causing a reduction in nociceptive thresholds and an increase in sensitivity (41). These pieces of evidence illustrate that 5-HT disorder is one of the overlapping pathogenesis mechanisms of MDD and migraine. Polymorphisms of the dopamine receptor gene affect the expression of dopamine receptors in the presynaptic membrane. It will affect the release, binding, and reuptake of dopamine in synapses (38). These alterations in synaptic transmission can contribute to the manifestation of symptoms associated with depression and migraine. Moreover, genetic polymorphisms in the methylenetetrahydrofolate reductase enzyme have been linked to migraine and MDD by impacting the development of cortical diffusion inhibition and disrupting methylation in the central nervous system, respectively (39).

According to a study of 109 patients with eating disorders (70% of AN patients and 30% of bulimia nervosa patients), it was found that 81 patients satisfied HIS criteria of migraine, with 55% experiencing migraine without aura and 2.8% experiencing migraine with aura. Additionally, 68% of the patients had a history of migraines before the onset of their eating disorder (10, 42). It aligned with our MR results that migraine without aura was associated with a high risk of AN. Although the pathogeny of eating disorders was still unclear, the plasma levels of dopamine, noradrenaline, tyrosine, and octopamine of patients with eating disorders or migraine were disorderly in comparison to those in healthy individuals (43, 44).

In contrast to conventional research methods, MR not only involved a substantial number of GWAS samples but also minimized the influence of confounding factors and reverse causality, allowing for a more precise assessment of the causal relationships between variables. Furthermore, some ethical issues that conventional research methods cannot avoid were circumvented by MR analysis. However, ethical considerations remain pertinent when working with genetic data, particularly regarding the privacy of patients whose samples are being utilized. Strict confidentiality must be upheld for personally identifiable information to prevent the unauthorized disclosure of patient personal data, which could lead to potential harassment or discrimination against patients and their families. Genetic advancements hold promise in managing genetic diseases and enhancing the overall public health for patients and their families. Nevertheless, it is imperative to address the question of distributive justice, ensuring that patients and their families have access to and can benefit from the research outcomes of genetic discoveries.

There were several limitations in our study. First, all GWAS data used in this study were derived from European ancestry, which may restrict the generalizability of our findings to other populations. Secondly, an arbitrary threshold of 1 × 10^−4^ was used to select SNPs for the sufficient number of IVs of migraine with aura and migraine without aura. Hence, some of the results of reverse MR were inevitably characterized by a level of pleiotropy. Thirdly, heterogeneity was detected in some results of reverse MR by Cochrane Q-test, but sensitivity analysis indicates that the results are relatively robust.

## 5 Conclusion

In conclusion, we found evidence that MDD was associated with a high risk of migraine without aura, but BIP was related to a low risk of migraine without aura. Migraine with aura was associated with a high risk of BIP and migraine without aura was associated with an increased risk of AN. Further research is warranted to discover the more specific mechanisms of psychiatric disorders and migraine. This study may provide new insight to prevent psychiatric disorders and migraine.

## Data availability statement

The original contributions presented in the study are included in the article/Supplementary material, further inquiries can be directed to the corresponding authors.

## Ethics statement

Ethical review and approval was not required for the study on human participants in accordance with the local legislation and institutional requirements. Written informed consent from the patients/participants or patients/participants' legal guardian/next of kin was not required to participate in this study in accordance with the national legislation and the institutional requirements.

## Author contributions

W-WL: Conceptualization, Data curation, Formal analysis, Investigation, Methodology, Project administration, Software, Supervision, Visualization, Writing – original draft, Writing – review & editing. J-XZ: Investigation, Methodology, Writing – review & editing. JW: Conceptualization, Data curation, Methodology, Writing – original draft. Y-qC: Investigation, Writing – review & editing. SL: Conceptualization, Data curation, Methodology, Software, Writing – review & editing. Z-KQ: Conceptualization, Methodology, Software, Writing – review & editing.

## Footnotes

^1^http://www.ebi.ac.uk/gwas

## Supplementary material

SUPPLEMENTARY TABLE S1 SNPs of psychiatric disorders and migraine.

SUPPLEMENTARY FIGURE S1 Funnel plot of SNPs. **(A)** Anorexia nervosa on migraine with aura, **(B)** bipolar disorder on migraine with aura, **(C)** major depressive disorder on migraine with aura, **(D)** post-traumatic stress disorder on migraine with aura, **(E)** schizophrenia on migraine with aura.

SUPPLEMENTARY FIGURE S2 Funnel plot of SNPs. **(A)** Anorexia nervosa on migraine without aura, **(B)** bipolar disorder on migraine without aura, **(C)** major depressive disorder on migraine without aura, **(D)** post-traumatic stress disorder on migraine without aura, **(E)** schizophrenia on migraine without aura.

SUPPLEMENTARY FIGURE S3 Forest plot of the causal effect of psychiatric disorders on migraine with aura and associated SNPs.

SUPPLEMENTARY FIGURE S4 Forest plot of the causal effect of psychiatric disorders on migraine without aura and associated SNPs.

SUPPLEMENTARY FIGURE S5 Funnel plot of SNPs. **(A)** Migraine with aura on anorexia nervosa, **(B)** migraine with aura on bipolar disorder, **(C)** migraine with aura on major depressive disorder, **(D)** migraine with aura on post-traumatic stress disorder, **(E)** migraine with aura on schizophrenia.

SUPPLEMENTARY FIGURE S6 Funnel plot of SNPs. **(A)** Migraine without aura on anorexia nervosa, **(B)** migraine without aura on bipolar disorder, **(C)** migraine without aura on major depressive disorder, **(D)** migraine without aura on post-traumatic stress disorder, **(E)** migraine without aura on schizophrenia.

SUPPLEMENTARY FIGURE S7 Forest plot of the causal effect of migraine with aura on psychiatric disorders and associated SNPs.

SUPPLEMENTARY FIGURE S8 Forest plot of the causal effect of migraine without aura on psychiatric disorders and associated SNPs.
