# Supplementary material for: Bidirectional two-sample Mendelian randomization analysis identifies causal associations between migraine and five psychiatric disorders
Source: Front Neurol. 2024 Aug 5;15:1432966. doi: 10.3389/fneur.2024.1432966 (PMC11330824; doi:10.3389/fneur.2024.1432966)
Supplement: SUPPLEMENTARY TABLE S1 — SNPs of psychiatric disorders and migraine. [file Table_1.DOCX]

Supplementary Materials Table 1:

Instrumental Variable

1. AN

| **SNP** | **EA** | **OA** | **Beta** | **SE** | **P-value** |
| --- | --- | --- | --- | --- | --- |
| rs10882687 | G | A | 0.0762 | 0.0166 | 4.24E-06 |
| rs112502176 | G | T | -0.0812 | 0.0171 | 1.97E-06 |
| rs11615526 | G | A | 0.0903 | 0.0172 | 1.52E-07 |
| rs12315996 | C | T | -0.0790 | 0.0170 | 3.19E-06 |
| rs12345267 | T | C | 0.0627 | 0.0136 | 4.28E-06 |
| rs12826213 | C | T | 0.0689 | 0.0138 | 6.21E-07 |
| rs13100344 | T | A | 0.0746 | 0.0136 | 4.21E-08 |
| rs13125932 | C | T | -0.0725 | 0.0134 | 5.85E-08 |
| rs13422947 | G | A | 0.0880 | 0.0190 | 3.50E-06 |
| rs1539725 | T | C | -0.0734 | 0.0139 | 1.26E-07 |
| rs17580614 | A | G | -0.0855 | 0.0186 | 4.41E-06 |
| rs17842856 | T | C | 0.0768 | 0.0168 | 4.95E-06 |
| rs189391781 | A | G | -0.239 | 0.0506 | 2.28E-06 |
| rs2008387 | G | A | -0.0815 | 0.0145 | 1.73E-08 |
| rs2131959 | G | C | 0.0771 | 0.0150 | 3.04E-07 |
| rs2287348 | C | T | -0.104 | 0.0179 | 5.62E-09 |
| rs2821359 | T | C | -0.0820 | 0.0161 | 3.76E-07 |
| rs2884594 | A | G | -0.0866 | 0.0177 | 9.37E-07 |
| rs34029884 | A | C | -0.0714 | 0.0152 | 2.43E-06 |
| rs34306607 | A | G | -0.0651 | 0.0138 | 2.53E-06 |
| rs35643176 | A | T | -0.125 | 0.0264 | 2.09E-06 |
| rs370838138 | G | C | 0.0753 | 0.0136 | 3.17E-08 |
| rs3848726 | G | T | 0.0658 | 0.0143 | 4.44E-06 |
| rs570333 | C | T | -0.0651 | 0.0139 | 2.82E-06 |
| rs6092932 | G | A | 0.0953 | 0.0187 | 3.50E-07 |
| rs61754806 | G | A | -0.156 | 0.0331 | 2.50E-06 |
| rs62176532 | T | C | 0.0856 | 0.0171 | 5.40E-07 |
| rs6520157 | G | A | -0.180 | 0.0380 | 2.12E-06 |
| rs6589488 | A | T | 0.127 | 0.0195 | 6.31E-11 |
| rs6773424 | G | A | -0.0910 | 0.0197 | 3.70E-06 |
| rs6789500 | T | C | 0.0908 | 0.0176 | 2.55E-07 |
| rs6792954 | G | A | 0.0761 | 0.0164 | 3.41E-06 |
| rs72635674 | T | C | -0.0798 | 0.0166 | 1.47E-06 |
| rs72734967 | G | A | -0.113 | 0.0236 | 1.66E-06 |
| rs7300592 | G | A | -0.0944 | 0.0202 | 2.80E-06 |
| rs7559141 | T | A | -0.294 | 0.0612 | 1.58E-06 |
| rs76453976 | A | G | -0.164 | 0.0359 | 4.53E-06 |
| rs7797895 | A | C | 0.0778 | 0.0165 | 2.33E-06 |
| rs9309871 | C | A | -0.0779 | 0.0158 | 8.58E-07 |
| rs9784437 | A | G | -0.0834 | 0.0169 | 8.01E-07 |
| rs9874207 | T | C | -0.0813 | 0.0145 | 2.05E-08 |
| rs9929084 | C | G | 0.0695 | 0.0149 | 2.99E-06 |

1. BIP

| **SNP** | **EA** | **OA** | **Beta** | **SE** | **P-value** |
| --- | --- | --- | --- | --- | --- |
| rs10255167 | G | A | -0.0664 | 0.0118 | 1.60E-08 |
| rs10455979 | C | G | -0.0556 | 0.00950 | 4.22E-09 |
| rs10737496 | C | T | 0.0542 | 0.00940 | 7.17E-09 |
| rs10791849 | T | A | 0.0694 | 0.0121 | 9.89E-09 |
| rs10866641 | T | C | 0.0626 | 0.00940 | 2.79E-11 |
| rs10994415 | T | C | -0.118 | 0.0174 | 1.14E-11 |
| rs115694474 | T | A | 0.0662 | 0.0119 | 2.35E-08 |
| rs11764361 | A | G | 0.0615 | 0.0104 | 3.47E-09 |
| rs12575685 | G | A | -0.0652 | 0.0101 | 1.24E-10 |
| rs13044225 | A | G | -0.0547 | 0.00950 | 8.50E-09 |
| rs17183814 | G | A | 0.103 | 0.0185 | 2.68E-08 |
| rs2011302 | T | A | -0.0529 | 0.00970 | 4.25E-08 |
| rs2273738 | C | T | -0.0917 | 0.0136 | 1.63E-11 |
| rs237460 | C | T | -0.0553 | 0.00940 | 4.25E-09 |
| rs28455634 | G | A | 0.0628 | 0.00990 | 2.63E-10 |
| rs28565152 | G | A | -0.0671 | 0.0112 | 1.96E-09 |
| rs2953928 | G | A | -0.116 | 0.0200 | 6.25E-09 |
| rs35958438 | G | A | 0.0642 | 0.0117 | 3.83E-08 |
| rs4331993 | T | A | -0.0547 | 0.00970 | 1.98E-08 |
| rs4447398 | A | C | 0.0822 | 0.0138 | 2.61E-09 |
| rs5758064 | T | C | 0.0524 | 0.00930 | 2.01E-08 |
| rs61554907 | G | T | -0.0868 | 0.0154 | 1.64E-08 |
| rs62489493 | C | G | -0.0897 | 0.0135 | 2.64E-11 |
| rs62581014 | C | T | -0.0652 | 0.0117 | 2.77E-08 |
| rs6946056 | A | C | -0.0532 | 0.00970 | 3.66E-08 |
| rs6954854 | G | A | 0.0583 | 0.00940 | 5.94E-10 |
| rs696366 | C | A | 0.0516 | 0.00940 | 4.46E-08 |
| rs7201930 | T | C | -0.0586 | 0.0104 | 1.89E-08 |
| rs7707252 | A | G | -0.0572 | 0.0104 | 3.64E-08 |

1. MDD

| **SNP** | **EA** | **OA** | **Beta** | **SE** | **P-value** |
| --- | --- | --- | --- | --- | --- |
| rs10913112 | T | C | -0.0262 | 0.00450 | 4.53E-09 |
| rs12919291 | C | G | 0.0327 | 0.00550 | 3.09E-09 |
| rs150186873 | A | C | -0.0704 | 0.0120 | 4.51E-09 |
| rs150346963 | T | C | 0.0283 | 0.00440 | 1.16E-10 |
| rs1931388 | A | G | 0.0295 | 0.00440 | 1.68E-11 |
| rs1950829 | A | G | 0.0297 | 0.00430 | 4.74E-12 |
| rs2214123 | A | G | 0.0261 | 0.00450 | 8.56E-09 |
| rs2247523 | C | G | -0.0243 | 0.00430 | 1.71E-08 |
| rs2418449 | T | C | 0.0281 | 0.00480 | 4.25E-09 |
| rs28541419 | C | G | 0.0292 | 0.00520 | 1.76E-08 |
| rs354155 | C | G | -0.0449 | 0.00750 | 1.75E-09 |
| rs4141983 | T | C | 0.0264 | 0.00460 | 9.69E-09 |
| rs508502 | T | C | -0.0264 | 0.00480 | 3.56E-08 |
| rs59082935 | T | C | 0.0363 | 0.00660 | 3.07E-08 |
| rs59283172 | A | G | -0.0390 | 0.00700 | 2.41E-08 |
| rs61914045 | A | G | 0.0309 | 0.00540 | 7.96E-09 |
| rs62535714 | A | G | 0.0339 | 0.00580 | 4.69E-09 |
| rs7551758 | T | G | -0.0283 | 0.00430 | 5.11E-11 |
| rs76954012 | A | T | 0.0412 | 0.00740 | 2.41E-08 |
| rs7725715 | A | G | 0.0290 | 0.00430 | 1.61E-11 |
| rs9364755 | A | G | -0.0283 | 0.00510 | 3.49E-08 |
| rs9529218 | T | C | -0.0340 | 0.00540 | 2.23E-10 |

1. PTSD

| **SNP** | **EA** | **OA** | **Beta** | **SE** | **P-value** |
| --- | --- | --- | --- | --- | --- |
| rs117405401 | A | G | 0.256 | 0.0553 | 3.69E-06 |
| rs1268149 | A | G | 0.380 | 0.0829 | 4.53E-06 |
| rs12706983 | T | G | -0.0896 | 0.0192 | 3.01E-06 |
| rs139591016 | T | C | -0.249 | 0.0532 | 2.86E-06 |
| rs140928208 | A | G | 0.447 | 0.0929 | 1.46E-06 |
| rs1444764 | A | G | 0.0781 | 0.0164 | 1.90E-06 |
| rs149509653 | A | G | 0.0905 | 0.0189 | 1.74E-06 |
| rs17108326 | A | G | 0.106 | 0.0209 | 3.91E-07 |
| rs2163050 | A | G | 0.100 | 0.0211 | 2.12E-06 |
| rs34517852 | A | T | 0.109 | 0.0185 | 3.16E-09 |
| rs36127550 | T | G | -0.103 | 0.0204 | 4.63E-07 |
| rs73154700 | A | G | 0.126 | 0.0272 | 3.45E-06 |
| rs763753 | A | G | -0.114 | 0.0221 | 2.43E-07 |
| rs77537694 | A | G | 0.130 | 0.0280 | 3.25E-06 |
| rs78608260 | A | G | -0.321 | 0.0694 | 3.71E-06 |

1. SCZ

| **SNP** | **EA** | **OA** | **Beta** | **SE** | **P-value** |
| --- | --- | --- | --- | --- | --- |
| rs10035564 | A | G | -0.0668 | 0.00920 | 4.38E-13 |
| rs10086619 | A | G | -0.0722 | 0.0116 | 4.97E-10 |
| rs10108980 | C | T | -0.0628 | 0.0106 | 2.73E-09 |
| rs10117 | G | A | 0.0550 | 0.00880 | 4.66E-10 |
| rs10861176 | G | A | -0.0555 | 0.00980 | 1.59E-08 |
| rs10876446 | G | C | -0.0540 | 0.00940 | 1.03E-08 |
| rs11027839 | A | C | -0.0515 | 0.00860 | 2.40E-09 |
| rs11136325 | G | A | 0.0538 | 0.00910 | 3.05E-09 |
| rs11165867 | C | T | -0.0743 | 0.0116 | 1.30E-10 |
| rs11223774 | A | G | 0.0525 | 0.00940 | 2.74E-08 |
| rs113264400 | T | C | -0.112 | 0.0202 | 2.87E-08 |
| rs11534045 | G | A | 0.0628 | 0.00930 | 1.40E-11 |
| rs11587347 | C | G | -0.104 | 0.0147 | 1.53E-12 |
| rs11693094 | C | T | 0.0544 | 0.00870 | 4.29E-10 |
| rs117178087 | C | T | 0.0964 | 0.0177 | 4.89E-08 |
| rs11941714 | G | A | 0.0516 | 0.00930 | 3.07E-08 |
| rs12129573 | C | A | -0.0778 | 0.00890 | 2.28E-18 |
| rs12138231 | T | A | -0.0670 | 0.0116 | 7.99E-09 |
| rs12293670 | A | G | 0.0705 | 0.00920 | 1.56E-14 |
| rs12303743 | G | C | -0.0875 | 0.0145 | 1.59E-09 |
| rs12652777 | T | C | 0.0488 | 0.00860 | 1.52E-08 |
| rs12712510 | T | C | 0.0574 | 0.00870 | 5.14E-11 |
| rs12771371 | G | A | 0.0524 | 0.00930 | 1.94E-08 |
| rs12877581 | G | C | -0.0596 | 0.00990 | 1.80E-09 |
| rs13016542 | T | C | 0.0883 | 0.0129 | 8.28E-12 |
| rs13233308 | C | T | 0.0487 | 0.00860 | 1.75E-08 |
| rs132582 | C | T | 0.0510 | 0.00860 | 3.26E-09 |
| rs1427633 | G | C | 0.0483 | 0.00880 | 4.10E-08 |
| rs1430894 | C | T | -0.0533 | 0.00860 | 6.15E-10 |
| rs145071536 | T | C | -0.0851 | 0.0120 | 1.62E-12 |
| rs149165 | T | G | 0.0482 | 0.00870 | 3.01E-08 |
| rs1540840 | G | C | 0.0557 | 0.00930 | 2.21E-09 |
| rs1593304 | A | G | -0.06410 | 0.0111 | 7.45E-09 |
| rs16851048 | T | C | -0.0745 | 0.0107 | 4.15E-12 |
| rs16867571 | A | G | 0.06570 | 0.0104 | 2.68E-10 |
| rs17016552 | C | G | 0.0517 | 0.00910 | 1.20E-08 |
| rs17731 | G | A | -0.0524 | 0.00890 | 4.37E-09 |
| rs1881046 | G | T | 0.0507 | 0.00920 | 3.39E-08 |
| rs1892346 | T | A | -0.0484 | 0.00880 | 3.56E-08 |
| rs1901512 | T | C | 0.0584 | 0.00940 | 5.72E-10 |
| rs1953205 | T | A | -0.0499 | 0.00890 | 2.22E-08 |
| rs2078266 | A | G | 0.0696 | 0.0126 | 2.94E-08 |
| rs215412 | G | A | -0.0577 | 0.00910 | 2.69E-10 |
| rs2238057 | T | G | -0.0835 | 0.00870 | 8.50E-22 |
| rs2252074 | T | G | -0.0685 | 0.00880 | 6.19E-15 |
| rs2333321 | A | G | 0.0712 | 0.0105 | 1.25E-11 |
| rs2381411 | T | C | -0.0504 | 0.00880 | 1.25E-08 |
| rs2455415 | C | T | -0.0495 | 0.00880 | 1.69E-08 |
| rs2532240 | C | T | 0.0608 | 0.00910 | 2.58E-11 |
| rs2999392 | C | T | -0.0518 | 0.00940 | 3.05E-08 |
| rs35351411 | A | C | -0.0635 | 0.00870 | 2.21E-13 |
| rs3739118 | G | A | 0.0570 | 0.00950 | 2.36E-09 |
| rs3791710 | T | C | 0.0600 | 0.0108 | 3.02E-08 |
| rs3824451 | T | C | -0.0656 | 0.0118 | 2.54E-08 |
| rs4575535 | A | G | -0.0558 | 0.00960 | 5.77E-09 |
| rs4766428 | C | T | -0.0750 | 0.00890 | 3.93E-17 |
| rs4779050 | T | G | 0.0580 | 0.00890 | 7.27E-11 |
| rs4812325 | G | A | -0.0719 | 0.00890 | 8.96E-16 |
| rs4921741 | A | G | -0.0560 | 0.00980 | 1.21E-08 |
| rs498591 | A | T | -0.0725 | 0.0121 | 2.11E-09 |
| rs500102 | T | C | 0.0517 | 0.00880 | 4.87E-09 |
| rs505061 | C | A | -0.0535 | 0.00860 | 5.80E-10 |
| rs56205728 | G | A | -0.0630 | 0.00970 | 1.01E-10 |
| rs56335113 | A | G | 0.0647 | 0.00940 | 6.02E-12 |
| rs57433322 | C | G | 0.0831 | 0.0139 | 1.99E-09 |
| rs5751191 | T | C | -0.0656 | 0.00860 | 3.00E-14 |
| rs58120505 | T | C | 0.0896 | 0.00880 | 2.24E-24 |
| rs6125656 | G | A | -0.0645 | 0.0111 | 6.29E-09 |
| rs62018952 | T | C | -0.0584 | 0.00970 | 1.94E-09 |
| rs62183855 | A | C | 0.0661 | 0.0111 | 2.66E-09 |
| rs634940 | G | T | -0.0664 | 0.00990 | 1.78E-11 |
| rs6482437 | A | C | -0.0989 | 0.0142 | 3.33E-12 |
| rs6538539 | G | T | 0.0568 | 0.00860 | 4.43E-11 |
| rs6549963 | T | C | 0.0483 | 0.00880 | 4.31E-08 |
| rs6673880 | A | G | -0.0623 | 0.00910 | 7.20E-12 |
| rs6715366 | G | A | -0.0541 | 0.00970 | 2.49E-08 |
| rs6798742 | A | G | -0.0611 | 0.00930 | 4.57E-11 |
| rs6943762 | T | C | 0.105 | 0.0132 | 1.57E-15 |
| rs6974218 | A | C | 0.0549 | 0.00890 | 6.80E-10 |
| rs6984242 | G | A | 0.0547 | 0.00870 | 3.86E-10 |
| rs713692 | G | A | -0.0566 | 0.00950 | 2.67E-09 |
| rs7251 | C | G | 0.0641 | 0.00940 | 8.29E-12 |
| rs72943392 | G | C | -0.0535 | 0.00960 | 2.39E-08 |
| rs73292401 | T | A | -0.0676 | 0.0109 | 5.48E-10 |
| rs7515363 | C | T | 0.0535 | 0.00890 | 1.84E-09 |
| rs7575796 | A | G | 0.0963 | 0.0172 | 2.07E-08 |
| rs7647398 | C | T | 0.0775 | 0.0109 | 1.07E-12 |
| rs76838079 | C | T | -0.0780 | 0.0138 | 1.53E-08 |
| rs7798283 | T | G | 0.0740 | 0.0134 | 3.49E-08 |
| rs7830315 | T | C | -0.0478 | 0.00860 | 3.08E-08 |
| rs79445414 | T | C | -0.123 | 0.0222 | 2.80E-08 |
| rs8055219 | G | A | -0.0665 | 0.0101 | 5.69E-11 |
| rs9304548 | C | A | 0.0567 | 0.0100 | 1.59E-08 |
| rs9318627 | A | C | 0.0612 | 0.00880 | 4.35E-12 |
| rs9876421 | C | T | -0.0625 | 0.00920 | 9.19E-12 |

1. non-aural migraine

| **SNP** | **EA** | **OA** | **Beta** | **SE** | **P-value** |
| --- | --- | --- | --- | --- | --- |
| rs10065659 | C | A | 0.115 | 0.0290 | 7.60E-05 |
| rs10778553 | G | T | -0.106 | 0.0261 | 4.65E-05 |
| rs11087103 | T | C | 0.169 | 0.0379 | 8.08E-06 |
| rs11096795 | A | C | 0.128 | 0.0306 | 3.06E-05 |
| rs111513989 | T | C | 0.158 | 0.0351 | 6.31E-06 |
| rs111756616 | A | G | 0.353 | 0.0899 | 8.72E-05 |
| rs11251880 | T | C | -0.116 | 0.0296 | 8.88E-05 |
| rs112961770 | T | G | 2.147 | 0.539 | 6.76E-05 |
| rs113050436 | G | T | -0.319 | 0.0783 | 4.56E-05 |
| rs113294655 | G | C | 0.267 | 0.0659 | 5.10E-05 |
| rs113344258 | A | G | -0.298 | 0.0763 | 9.14E-05 |
| rs1155688 | A | G | -0.148 | 0.0312 | 2.10E-06 |
| rs115668283 | C | T | 0.353 | 0.0804 | 1.10E-05 |
| rs11681583 | G | A | 0.129 | 0.0280 | 4.35E-06 |
| rs116898306 | A | C | 0.186 | 0.0443 | 2.78E-05 |
| rs117171962 | G | C | 0.184 | 0.0436 | 2.44E-05 |
| rs117872033 | T | C | 0.376 | 0.0895 | 2.62E-05 |
| rs12108081 | T | C | 0.130 | 0.0297 | 1.29E-05 |
| rs12152312 | A | G | -0.137 | 0.0329 | 3.01E-05 |
| rs12407041 | T | C | 0.160 | 0.0399 | 6.27E-05 |
| rs12418585 | A | G | 0.116 | 0.0293 | 7.12E-05 |
| rs12520244 | C | G | -0.157 | 0.0402 | 9.51E-05 |
| rs12762800 | T | C | 0.206 | 0.0491 | 2.82E-05 |
| rs12767952 | A | G | 0.177 | 0.0432 | 4.24E-05 |
| rs13215358 | G | A | 0.169 | 0.0395 | 1.85E-05 |
| rs13230396 | A | G | 0.138 | 0.0310 | 8.13E-06 |
| rs13391347 | T | G | -0.237 | 0.0602 | 7.99E-05 |
| rs13434366 | A | G | 0.107 | 0.0262 | 4.46E-05 |
| rs1355588 | A | G | 0.169 | 0.0418 | 5.42E-05 |
| rs139590715 | T | C | 0.478 | 0.122 | 8.96E-05 |
| rs1470998 | A | C | 0.317 | 0.0758 | 2.86E-05 |
| rs147720341 | A | G | 0.356 | 0.0882 | 5.40E-05 |
| rs149543195 | A | G | 0.250 | 0.0587 | 1.98E-05 |
| rs1532923 | T | C | 0.267 | 0.0675 | 7.59E-05 |
| rs1556010 | G | A | 0.105 | 0.0267 | 8.32E-05 |
| rs1577816 | C | G | -0.135 | 0.0311 | 1.39E-05 |
| rs158775 | C | T | -0.126 | 0.0303 | 3.04E-05 |
| rs17115602 | C | T | -0.177 | 0.0435 | 4.92E-05 |
| rs17574102 | G | A | -0.201 | 0.0514 | 8.76E-05 |
| rs17699863 | G | A | 0.183 | 0.0440 | 3.22E-05 |
| rs2167474 | C | T | -0.114 | 0.0285 | 6.35E-05 |
| rs2171492 | T | G | 0.115 | 0.0271 | 2.08E-05 |
| rs2191685 | C | G | 0.126 | 0.0288 | 1.24E-05 |
| rs228229 | G | C | -0.140 | 0.0351 | 6.70E-05 |
| rs2348845 | C | A | 0.106 | 0.0261 | 5.19E-05 |
| rs2460257 | A | C | 0.355 | 0.0852 | 3.06E-05 |
| rs251405 | C | T | -0.157 | 0.0398 | 8.04E-05 |
| rs2860262 | G | C | -0.122 | 0.0312 | 9.60E-05 |
| rs34308810 | T | A | -0.103 | 0.0265 | 9.92E-05 |
| rs343431 | A | G | 0.128 | 0.0315 | 4.47E-05 |
| rs34918172 | G | A | 0.106 | 0.0268 | 6.90E-05 |
| rs35045982 | T | G | 0.155 | 0.0396 | 8.82E-05 |
| rs35233611 | T | C | 0.478 | 0.107 | 8.26E-06 |
| rs35835084 | C | T | -0.111 | 0.0269 | 3.46E-05 |
| rs35874773 | G | A | 0.193 | 0.0485 | 7.07E-05 |
| rs35954094 | C | T | 0.739 | 0.184 | 5.70E-05 |
| rs372738957 | G | C | 0.176 | 0.0423 | 3.20E-05 |
| rs3743145 | C | A | -0.146 | 0.0361 | 4.95E-05 |
| rs3768377 | G | A | 0.138 | 0.0344 | 5.88E-05 |
| rs3821320 | G | A | 0.109 | 0.0273 | 6.42E-05 |
| rs4612935 | T | G | 0.143 | 0.0309 | 3.38E-06 |
| rs4740888 | C | T | -0.103 | 0.0262 | 7.72E-05 |
| rs61747149 | A | G | 0.827 | 0.208 | 7.20E-05 |
| rs61778163 | A | G | 0.112 | 0.0280 | 5.73E-05 |
| rs61980275 | A | G | 0.180 | 0.0399 | 6.73E-06 |
| rs620797 | T | A | 0.109 | 0.0276 | 8.54E-05 |
| rs62271153 | T | C | -0.325 | 0.0830 | 9.19E-05 |
| rs6443445 | C | T | -0.118 | 0.0286 | 3.71E-05 |
| rs6490179 | C | T | 0.121 | 0.0266 | 5.56E-06 |
| rs6662887 | T | C | 0.113 | 0.0286 | 8.00E-05 |
| rs6912271 | T | C | 0.164 | 0.0416 | 7.97E-05 |
| rs6944957 | A | G | 0.129 | 0.0284 | 5.37E-06 |
| rs7130497 | T | C | -0.113 | 0.0273 | 3.45E-05 |
| rs71370493 | T | C | 0.134 | 0.0306 | 1.10E-05 |
| rs71625651 | T | C | 0.171 | 0.0413 | 3.46E-05 |
| rs73145783 | G | A | 0.204 | 0.0392 | 2.03E-07 |
| rs73199934 | C | T | 0.224 | 0.0504 | 8.43E-06 |
| rs75151067 | A | G | 0.615 | 0.151 | 4.59E-05 |
| rs75280455 | A | C | 0.430 | 0.0942 | 5.08E-06 |
| rs75306382 | A | C | 0.222 | 0.0528 | 2.61E-05 |
| rs75866611 | T | C | 0.669 | 0.164 | 4.24E-05 |
| rs76191407 | A | T | 0.313 | 0.0725 | 1.63E-05 |
| rs7702582 | C | T | 0.161 | 0.0391 | 3.73E-05 |
| rs8047983 | C | T | 0.133 | 0.0293 | 6.00E-06 |
| rs9559035 | A | T | 0.102 | 0.0260 | 9.46E-05 |
| rs9854922 | C | T | 0.109 | 0.0272 | 6.41E-05 |
| rs10853269 | T | C | 0.122 | 0.0309 | 8.30E-05 |
| rs111733890 | T | C | -0.458 | 0.114 | 5.64E-05 |
| rs111823172 | T | C | 1.04 | 0.218 | 1.98E-06 |
| rs112253950 | A | G | 0.347 | 0.0761 | 5.30E-06 |
| rs116316096 | A | G | 0.360 | 0.0860 | 2.93E-05 |
| rs11768262 | A | C | -0.178 | 0.0379 | 2.85E-06 |
| rs11902122 | A | G | 0.110 | 0.0264 | 3.41E-05 |
| rs142947818 | A | G | 0.427 | 0.110 | 9.96E-05 |
| rs143024621 | A | G | 0.385 | 0.0985 | 9.23E-05 |
| rs145184423 | G | C | 0.287 | 0.0696 | 3.77E-05 |
| rs145912479 | G | A | 1.16 | 0.274 | 2.27E-05 |
| rs148177573 | T | A | 0.692 | 0.176 | 8.07E-05 |
| rs148763221 | T | G | 0.760 | 0.179 | 2.22E-05 |
| rs17546779 | C | T | 0.193 | 0.0480 | 5.58E-05 |
| rs192785708 | G | T | 0.355 | 0.0904 | 8.51E-05 |
| rs34583461 | C | A | 0.464 | 0.101 | 4.97E-06 |
| rs61491331 | A | G | 0.225 | 0.0496 | 5.58E-06 |
| rs61979240 | C | A | 0.446 | 0.112 | 7.25E-05 |
| rs67231891 | A | T | 0.481 | 0.119 | 5.49E-05 |
| rs74925396 | G | A | 0.399 | 0.0818 | 1.10E-06 |
| rs74986743 | C | T | 0.502 | 0.128 | 8.78E-05 |
| rs75116187 | A | C | 0.522 | 0.122 | 1.85E-05 |
| rs7527664 | G | C | 0.135 | 0.0338 | 6.56E-05 |
| rs76757807 | G | C | 0.374 | 0.0816 | 4.67E-06 |
| rs77944969 | A | G | -0.383 | 0.0887 | 1.59E-05 |
| rs9301946 | G | A | 0.596 | 0.149 | 6.64E-05 |
| rs9641113 | G | A | 0.117 | 0.0269 | 1.35E-05 |
| rs112877175 | C | A | 0.272 | 0.0642 | 2.28E-05 |
| rs116071613 | A | G | 1.51 | 0.340 | 9.12E-06 |
| rs117848382 | T | G | -0.396 | 0.094 | 2.82E-05 |
| rs9547464 | A | T | 0.790 | 0.1990 | 7.16E-05 |
| rs191973610 | T | C | 0.993 | 0.249 | 6.89E-05 |
| rs78631834 | G | A | 0.236 | 0.0574 | 4.07E-05 |

1. aural migraine

| **SNP** | **EA** | **OA** | **Beta** | **SE** | **P-value** |
| --- | --- | --- | --- | --- | --- |
| rs10008442 | A | G | -0.116 | 0.0291 | 6.28E-05 |
| rs10056028 | G | A | -0.102 | 0.0250 | 4.17E-05 |
| rs1011339 | C | A | -0.104 | 0.0250 | 3.24E-05 |
| rs11031133 | C | T | 0.111 | 0.0275 | 5.20E-05 |
| rs113183182 | A | G | 0.276 | 0.0661 | 3.04E-05 |
| rs114452453 | T | C | -0.282 | 0.0691 | 4.55E-05 |
| rs114738703 | C | A | -0.305 | 0.0715 | 2.03E-05 |
| rs115660862 | T | C | 0.277 | 0.0696 | 6.77E-05 |
| rs115978903 | G | A | 0.353 | 0.0838 | 2.58E-05 |
| rs117246882 | C | T | -0.171 | 0.0417 | 4.10E-05 |
| rs117269308 | T | G | 0.347 | 0.0858 | 5.37E-05 |
| rs117320778 | T | G | 1.15 | 0.288 | 6.20E-05 |
| rs11751200 | T | G | 0.297 | 0.0733 | 5.01E-05 |
| rs117744613 | G | C | 0.294 | 0.0718 | 4.18E-05 |
| rs1187655 | C | T | -0.117 | 0.0289 | 4.98E-05 |
| rs12124939 | G | A | 0.178 | 0.0451 | 7.84E-05 |
| rs1227755 | T | C | 0.304 | 0.0747 | 4.59E-05 |
| rs12359946 | C | T | 0.223 | 0.0568 | 8.89E-05 |
| rs12469472 | A | C | 2.22 | 0.540 | 3.95E-05 |
| rs12621276 | A | G | 0.129 | 0.0307 | 2.74E-05 |
| rs12775018 | T | C | 0.179 | 0.0452 | 7.66E-05 |
| rs139699059 | C | T | 0.562 | 0.124 | 5.50E-06 |
| rs146206669 | T | A | 0.334 | 0.0853 | 8.98E-05 |
| rs146240626 | T | C | 0.132 | 0.0290 | 5.40E-06 |
| rs147113296 | A | G | 0.285 | 0.0696 | 4.28E-05 |
| rs1497383 | A | G | -0.105 | 0.0249 | 2.40E-05 |
| rs1538633 | A | G | -0.108 | 0.0257 | 2.54E-05 |
| rs1538972 | A | G | -0.164 | 0.0375 | 1.22E-05 |
| rs1544862 | A | G | 0.127 | 0.0299 | 2.03E-05 |
| rs16035 | G | T | -0.128 | 0.0248 | 2.45E-07 |
| rs16844292 | C | T | 0.188 | 0.0469 | 6.11E-05 |
| rs17357335 | G | A | -0.175 | 0.0450 | 9.88E-05 |
| rs17522977 | T | G | 0.171 | 0.0426 | 5.94E-05 |
| rs189000965 | T | C | 0.650 | 0.164 | 7.52E-05 |
| rs189692316 | T | C | 0.444 | 0.110 | 5.75E-05 |
| rs192453758 | G | A | 0.194 | 0.0478 | 4.75E-05 |
| rs2160115 | T | C | 0.117 | 0.0269 | 1.37E-05 |
| rs2355017 | T | C | 0.118 | 0.0277 | 2.01E-05 |
| rs2455889 | T | G | -0.109 | 0.0265 | 3.71E-05 |
| rs2461043 | G | A | 0.102 | 0.0246 | 3.66E-05 |
| rs2917816 | T | C | 0.0978 | 0.0251 | 9.80E-05 |
| rs2993807 | T | C | 0.144 | 0.0359 | 6.21E-05 |
| rs34136224 | G | A | -0.102 | 0.0258 | 7.85E-05 |
| rs34910937 | G | C | -0.105 | 0.0248 | 2.12E-05 |
| rs35792437 | T | C | 0.129 | 0.0316 | 4.74E-05 |
| rs35958318 | A | G | 0.188 | 0.0476 | 8.15E-05 |
| rs36010444 | A | G | 0.111 | 0.0272 | 4.55E-05 |
| rs36215052 | T | G | 0.266 | 0.0630 | 2.51E-05 |
| rs3749742 | G | C | -0.118 | 0.0294 | 5.45E-05 |
| rs382583 | C | T | 0.164 | 0.0360 | 4.87E-06 |
| rs4483657 | G | A | 0.119 | 0.0296 | 5.63E-05 |
| rs4849052 | C | T | -0.535 | 0.102 | 1.49E-07 |
| rs4879993 | T | C | 0.103 | 0.0261 | 8.57E-05 |
| rs4920616 | G | T | -0.231 | 0.0546 | 2.22E-05 |
| rs55838133 | C | A | 0.310 | 0.0789 | 8.46E-05 |
| rs58553282 | A | G | 0.235 | 0.0545 | 1.55E-05 |
| rs61857306 | A | C | 0.184 | 0.0443 | 3.32E-05 |
| rs62389343 | C | T | 0.506 | 0.128 | 7.85E-05 |
| rs62457050 | T | C | 0.175 | 0.0376 | 3.10E-06 |
| rs62492683 | G | A | 0.128 | 0.0320 | 6.58E-05 |
| rs6539333 | C | T | 0.103 | 0.0264 | 9.72E-05 |
| rs67497653 | T | A | 0.107 | 0.0250 | 1.89E-05 |
| rs6987343 | A | C | -0.103 | 0.0247 | 2.93E-05 |
| rs7091513 | T | C | 0.150 | 0.0354 | 2.26E-05 |
| rs7135461 | A | C | 0.120 | 0.0247 | 1.36E-06 |
| rs72727816 | G | A | -0.164 | 0.0415 | 8.20E-05 |
| rs73004193 | G | A | 0.339 | 0.0717 | 2.29E-06 |
| rs73199947 | A | G | 0.154 | 0.0375 | 4.03E-05 |
| rs73928973 | C | A | 0.200 | 0.0447 | 7.33E-06 |
| rs7442659 | A | C | 0.116 | 0.0288 | 5.25E-05 |
| rs7452764 | G | A | 0.395 | 0.0901 | 1.18E-05 |
| rs748245 | A | T | -0.128 | 0.0328 | 9.81E-05 |
| rs75040810 | A | G | -0.350 | 0.0879 | 7.03E-05 |
| rs7517714 | T | C | -0.112 | 0.0265 | 2.23E-05 |
| rs76497810 | T | C | 0.591 | 0.146 | 5.27E-05 |
| rs76828150 | C | T | 0.612 | 0.146 | 2.71E-05 |
| rs7724203 | C | T | 0.144 | 0.0324 | 9.54E-06 |
| rs7792601 | T | C | 0.132 | 0.0325 | 4.71E-05 |
| rs77930149 | A | C | 0.254 | 0.0577 | 1.08E-05 |
| rs7824222 | C | G | 0.186 | 0.0471 | 7.98E-05 |
| rs78455122 | A | G | 0.116 | 0.0293 | 7.88E-05 |
| rs892649 | C | T | -0.111 | 0.0278 | 6.70E-05 |
| rs9309304 | C | T | 0.104 | 0.0256 | 5.17E-05 |
| rs9369281 | C | A | -0.117 | 0.0294 | 7.04E-05 |
| rs9400094 | C | T | 0.137 | 0.0340 | 5.83E-05 |
| rs953255 | C | A | 0.173 | 0.0387 | 7.79E-06 |
| rs9534239 | C | T | -0.150 | 0.0348 | 1.63E-05 |
| rs9827613 | G | A | 0.0985 | 0.0246 | 6.36E-05 |
| rs9913843 | G | A | 0.180 | 0.0416 | 1.53E-05 |
| rs9991854 | A | C | -0.117 | 0.0292 | 6.36E-05 |
| rs112790169 | T | C | 0.611 | 0.142 | 1.73E-05 |
| rs114027538 | G | A | 0.516 | 0.121 | 2.12E-05 |
| rs115865728 | A | T | 0.565 | 0.140 | 5.38E-05 |
| rs117208805 | A | T | -0.207 | 0.0497 | 3.08E-05 |
| rs117287758 | C | T | -0.201 | 0.0460 | 1.31E-05 |
| rs117750534 | T | A | -0.303 | 0.0773 | 8.90E-05 |
| rs117970501 | G | A | -0.290 | 0.0725 | 6.12E-05 |
| rs140705160 | T | C | -0.138 | 0.0354 | 9.67E-05 |
| rs142567494 | T | C | 0.268 | 0.0644 | 3.26E-05 |
| rs142937471 | T | C | 0.434 | 0.0973 | 8.08E-06 |
| rs144755021 | A | G | 0.532 | 0.119 | 8.01E-06 |
| rs146820126 | A | G | 0.191 | 0.0487 | 8.59E-05 |
| rs147452852 | C | T | 0.614 | 0.151 | 4.75E-05 |
| rs156217 | C | T | -0.366 | 0.0936 | 9.29E-05 |
| rs17271445 | G | T | -0.188 | 0.0423 | 8.32E-06 |
| rs186399358 | T | C | -0.325 | 0.0805 | 5.53E-05 |
| rs186680879 | G | A | 0.551 | 0.127 | 1.42E-05 |
| rs2104196 | A | G | 0.0994 | 0.0255 | 9.54E-05 |
| rs2385826 | A | G | -0.126 | 0.0313 | 6.16E-05 |
| rs2412067 | G | A | -0.104 | 0.0259 | 6.05E-05 |
| rs34897924 | G | A | 0.133 | 0.0308 | 1.47E-05 |
| rs35011334 | C | T | -0.109 | 0.0265 | 4.10E-05 |
| rs41272845 | A | G | 0.286 | 0.0720 | 7.27E-05 |
| rs45528036 | T | C | 0.643 | 0.155 | 3.30E-05 |
| rs4968175 | C | T | 0.392 | 0.0931 | 2.51E-05 |
| rs754567 | G | T | -0.112 | 0.0261 | 1.79E-05 |
| rs77999946 | G | A | -0.257 | 0.0630 | 4.48E-05 |
| rs113292455 | A | C | 0.691 | 0.161 | 1.79E-05 |
| rs117489824 | A | C | -0.492 | 0.126 | 9.11E-05 |
| rs184414116 | A | G | 1.77 | 0.427 | 3.52E-05 |
| rs189638748 | A | G | 0.661 | 0.144 | 4.19E-06 |
| rs73131517 | C | G | 0.278 | 0.0674 | 3.55E-05 |
| rs115882822 | G | C | 0.711 | 0.181 | 8.29E-05 |
