# Supplementary figures and images for: Bidirectional two-sample Mendelian randomization analysis identifies causal associations between migraine and five psychiatric disorders
Source: Front Neurol. 2024 Aug 5;15:1432966. doi: 10.3389/fneur.2024.1432966 (PMC11330824; doi:10.3389/fneur.2024.1432966)

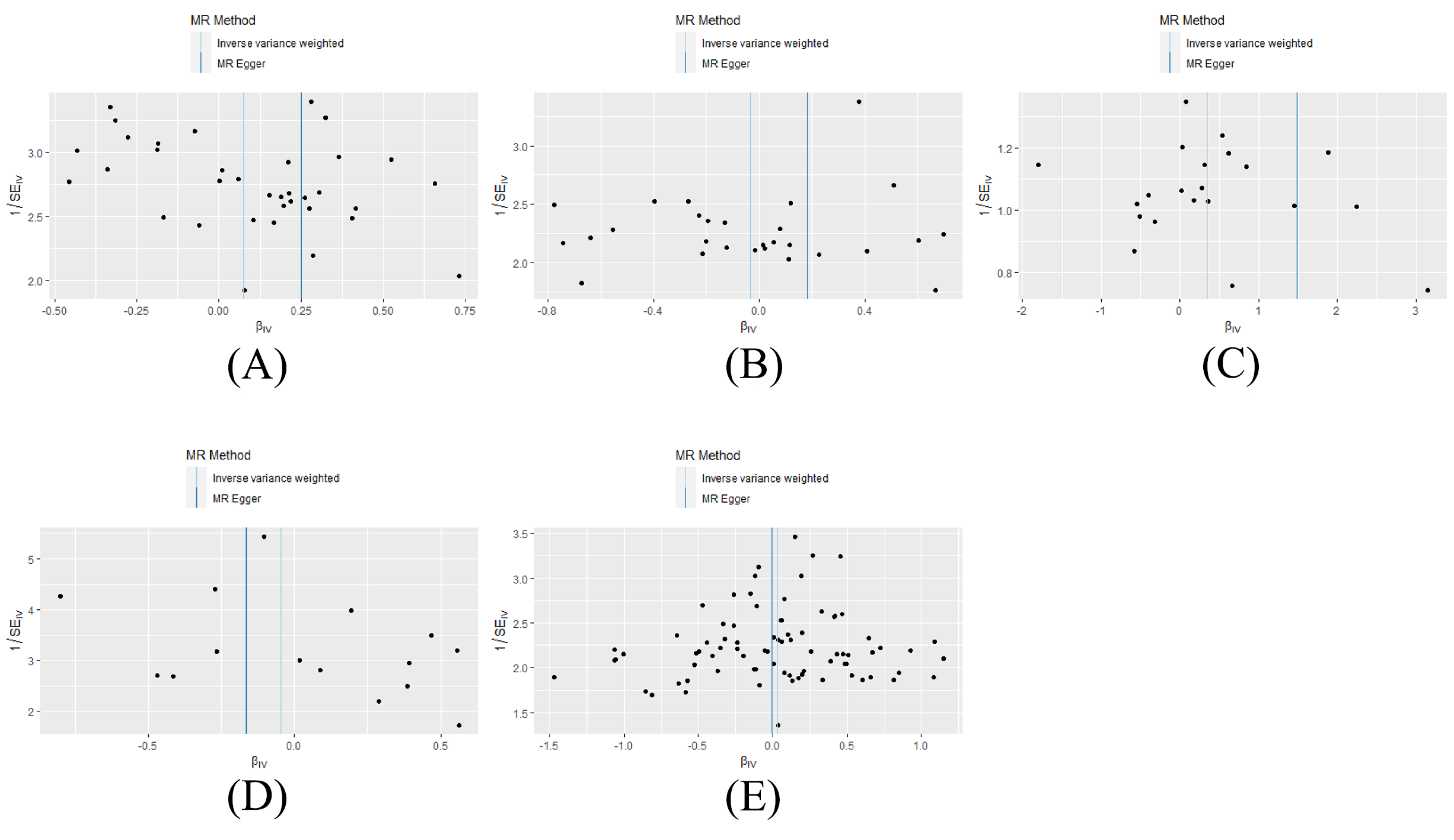

Supplement: SUPPLEMENTARY FIGURE S1 — Funnel plot of SNPs. (A) Anorexia nervosa on migraine with aura, (B) bipolar disorder on migraine with aura, (C) major depressive disorder on migraine with aura, (D) post-traumatic stress disorder on migraine with aura, (E) schizophrenia on migraine with aura. [file Image_1.TIF]

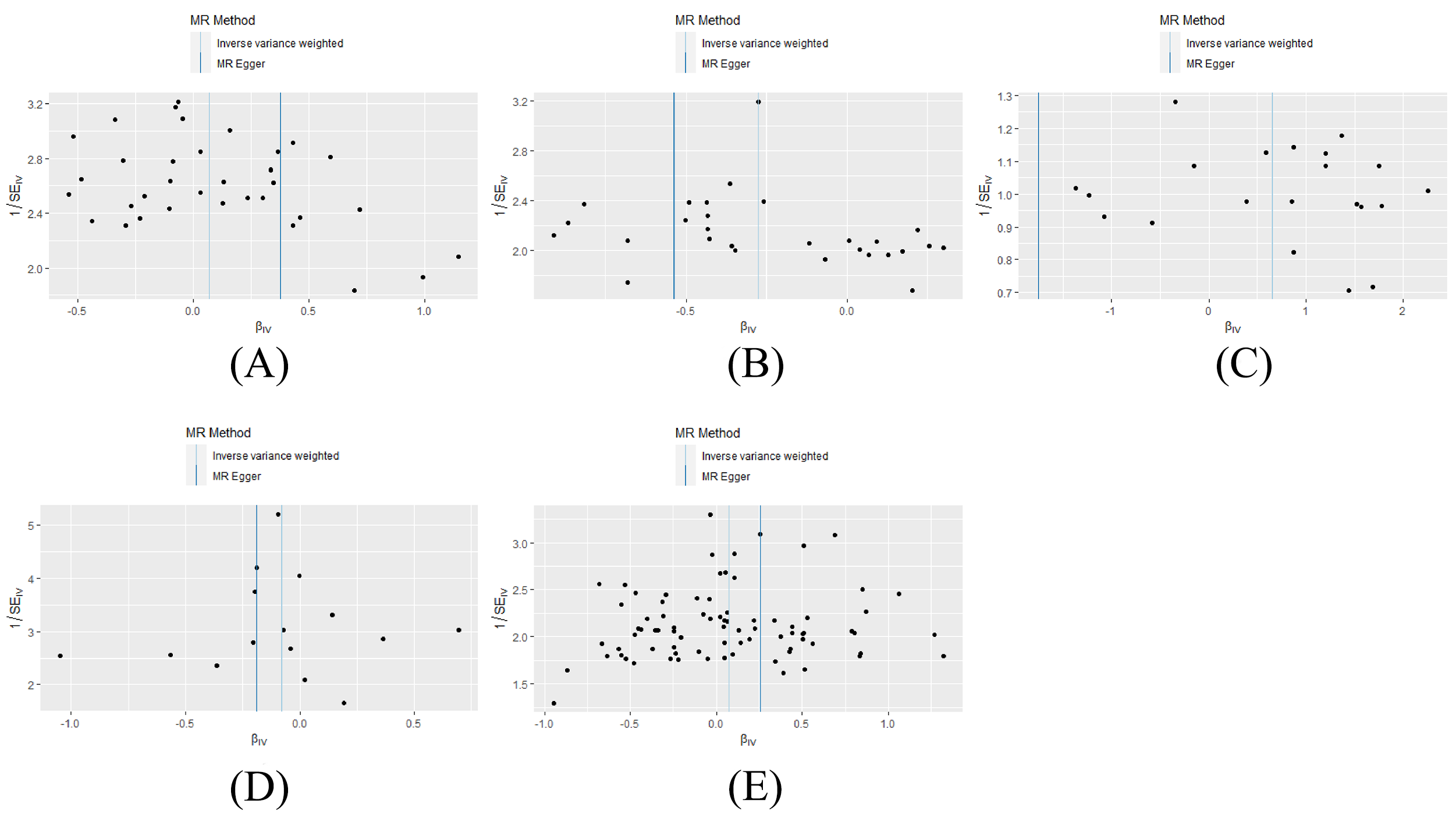

Supplement: SUPPLEMENTARY FIGURE S2 — Funnel plot of SNPs. (A) Anorexia nervosa on migraine without aura, (B) bipolar disorder on migraine without aura, (C) major depressive disorder on migraine without aura, (D) post-traumatic stress disorder on migraine without aura, (E) schizophrenia on migraine without aura. [file Image_2.TIF]

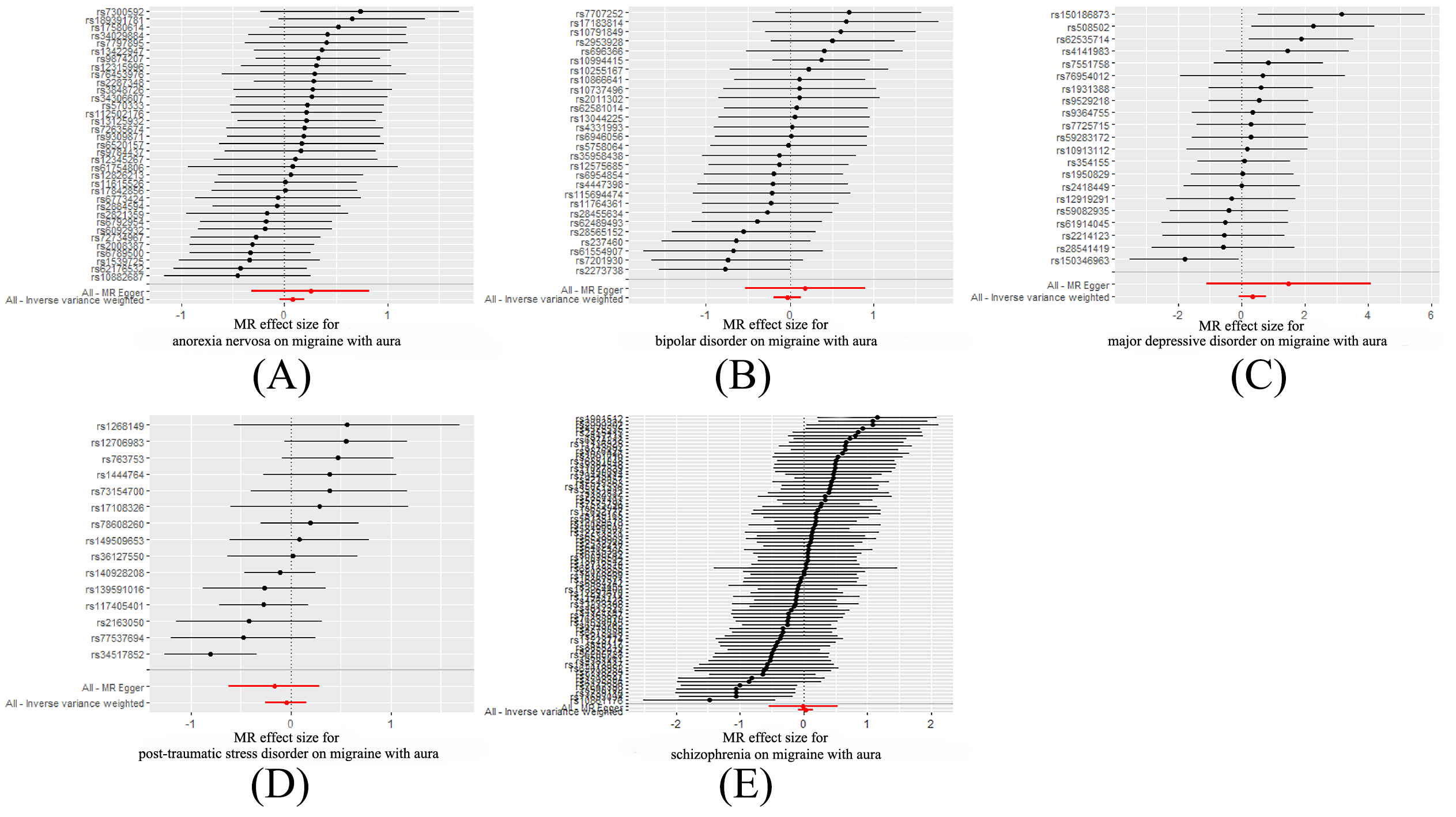

Supplement: SUPPLEMENTARY FIGURE S3 — Forest plot of the causal effect of psychiatric disorders on migraine with aura and associated SNPs. [file Image_3.TIF]

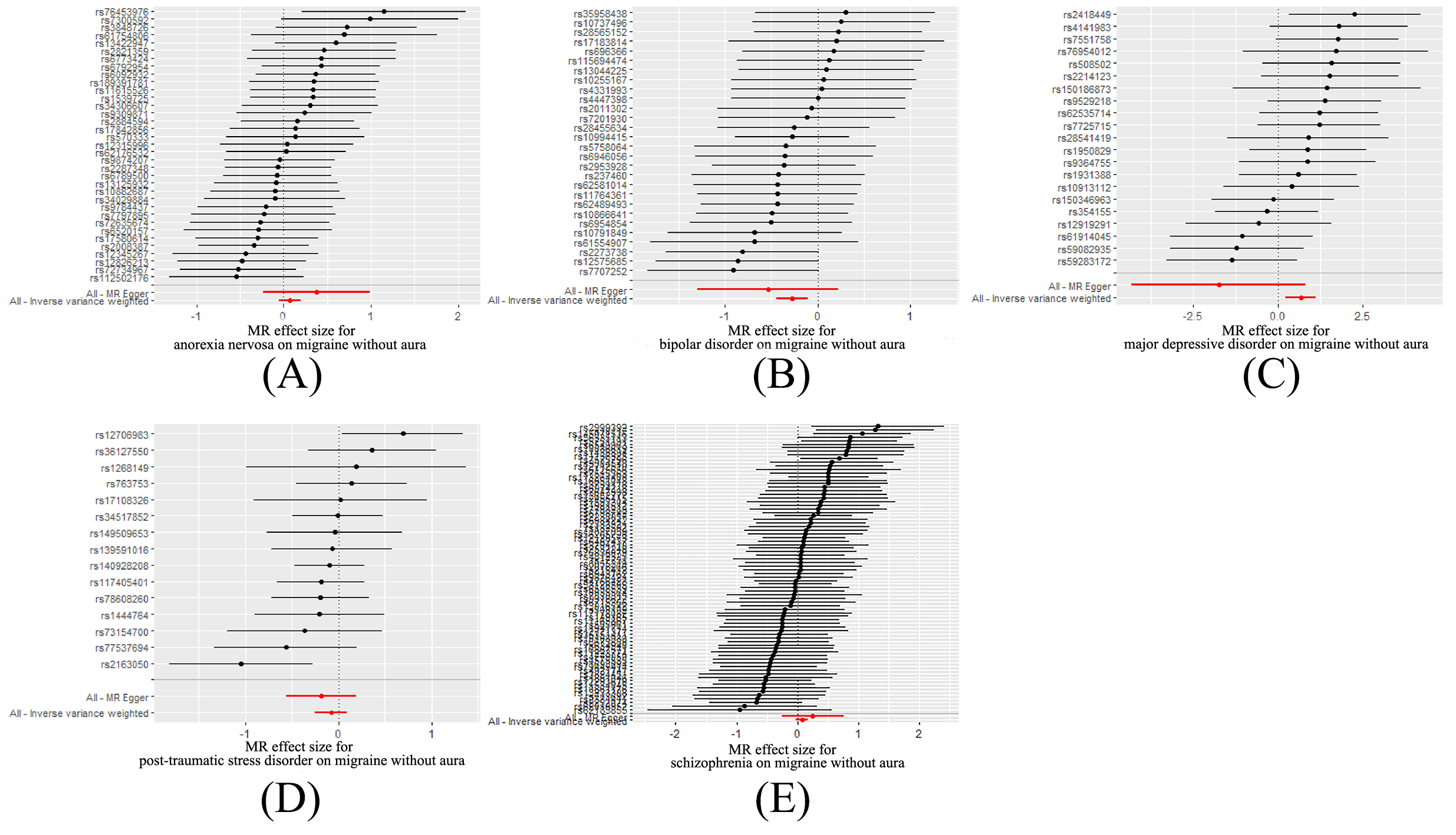

Supplement: SUPPLEMENTARY FIGURE S4 — Forest plot of the causal effect of psychiatric disorders on migraine without aura and associated SNPs. [file Image_4.TIF]

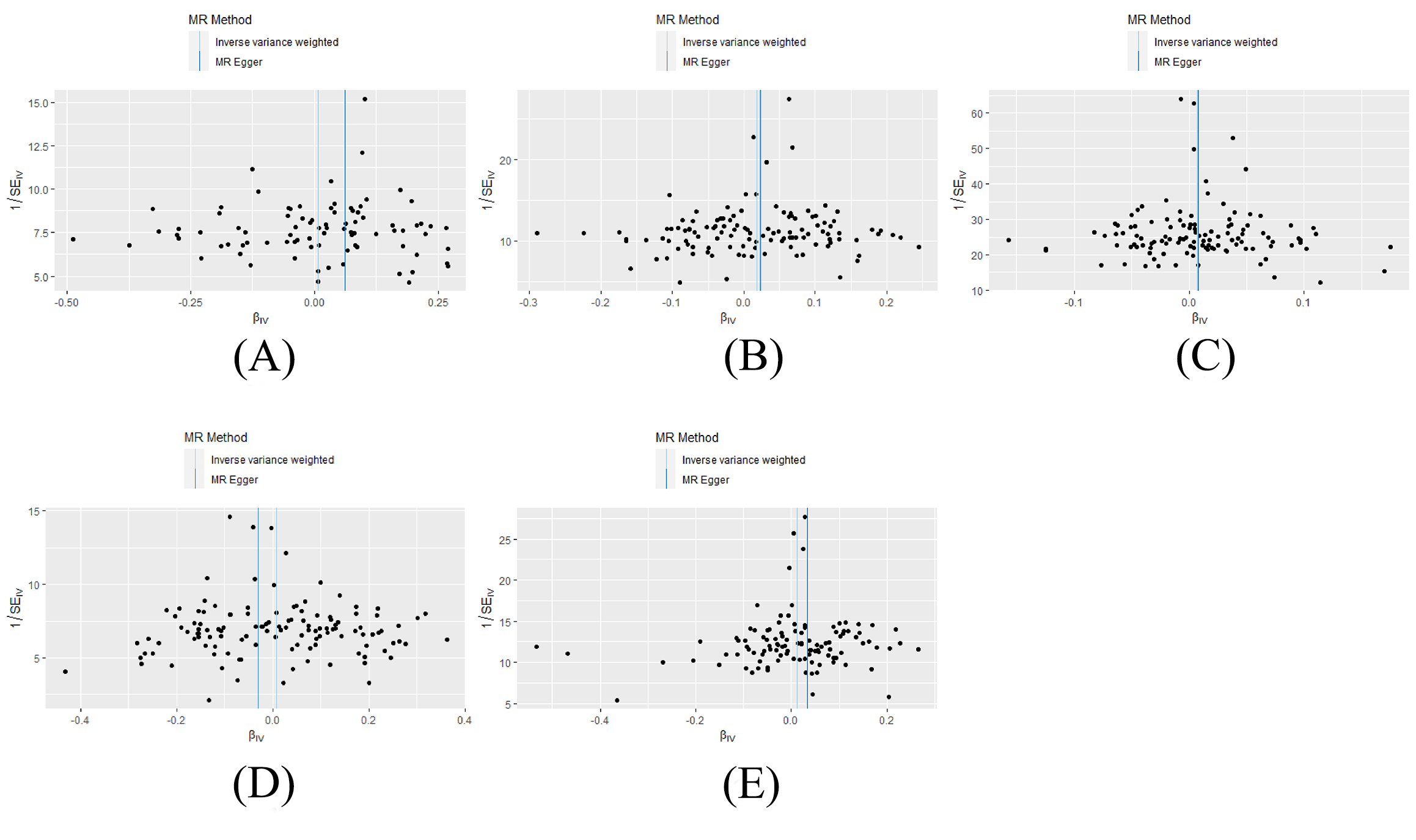

Supplement: SUPPLEMENTARY FIGURE S5 — Funnel plot of SNPs. (A) Migraine with aura on anorexia nervosa, (B) migraine with aura on bipolar disorder, (C) migraine with aura on major depressive disorder, (D) migraine with aura on post-traumatic stress disorder, (E) migraine with aura on schizophrenia. [file Image_5.TIF]

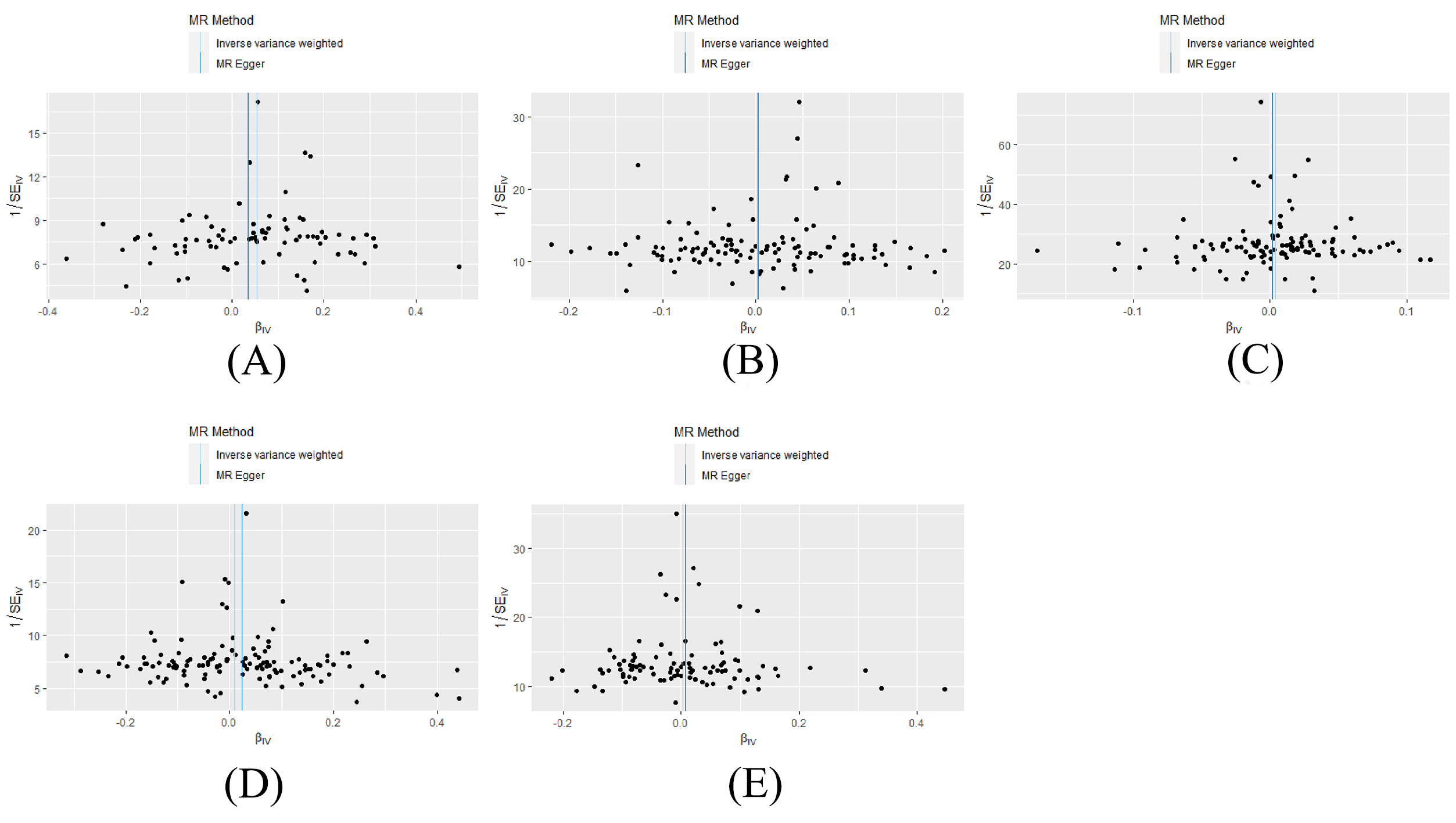

Supplement: SUPPLEMENTARY FIGURE S6 — Funnel plot of SNPs. (A) Migraine without aura on anorexia nervosa, (B) migraine without aura on bipolar disorder, (C) migraine without aura on major depressive disorder, (D) migraine without aura on post-traumatic stress disorder, (E) migraine without aura on schizophrenia. [file Image_6.TIF]

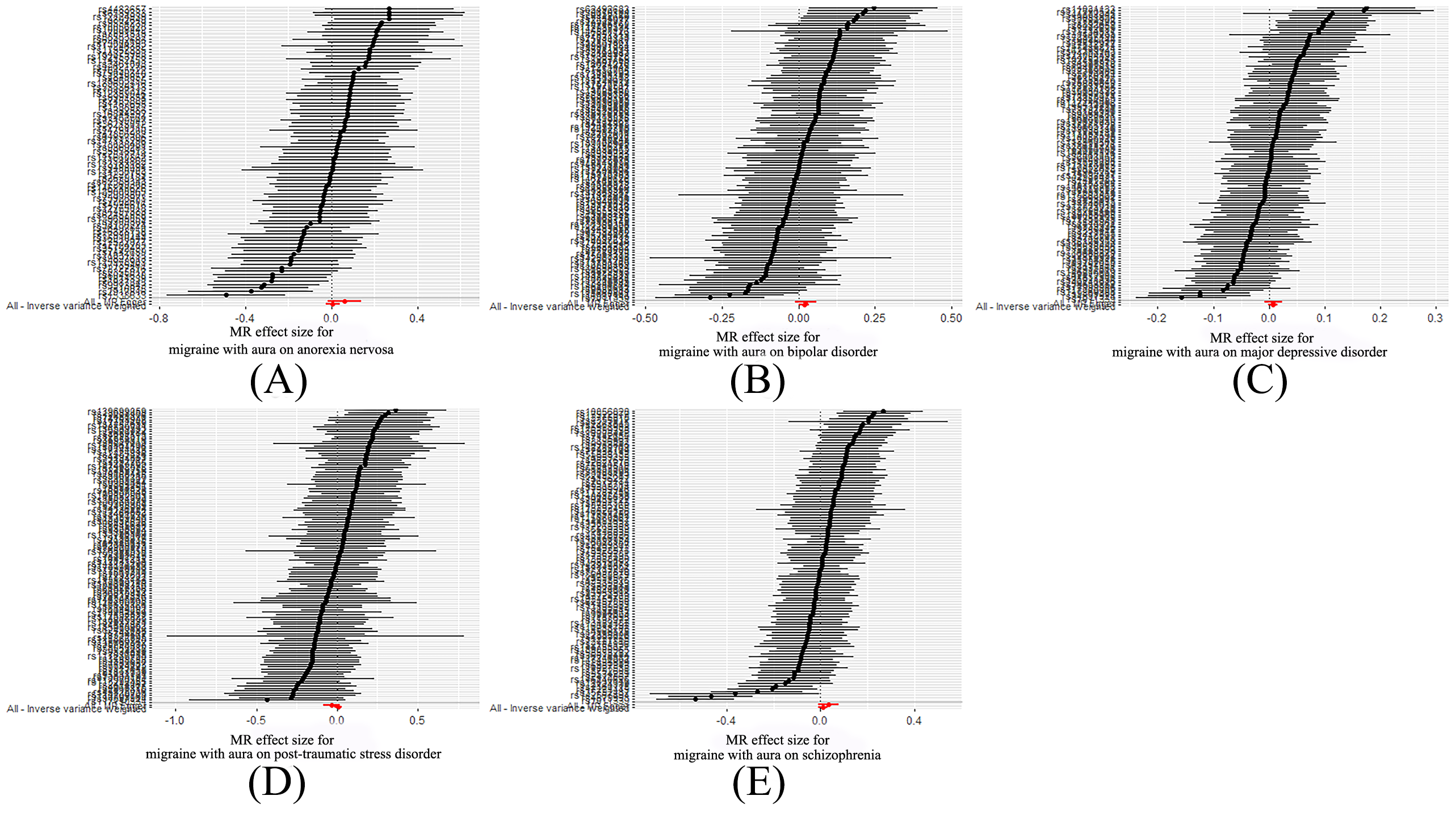

Supplement: SUPPLEMENTARY FIGURE S7 — Forest plot of the causal effect of migraine with aura on psychiatric disorders and associated SNPs. [file Image_7.TIF]

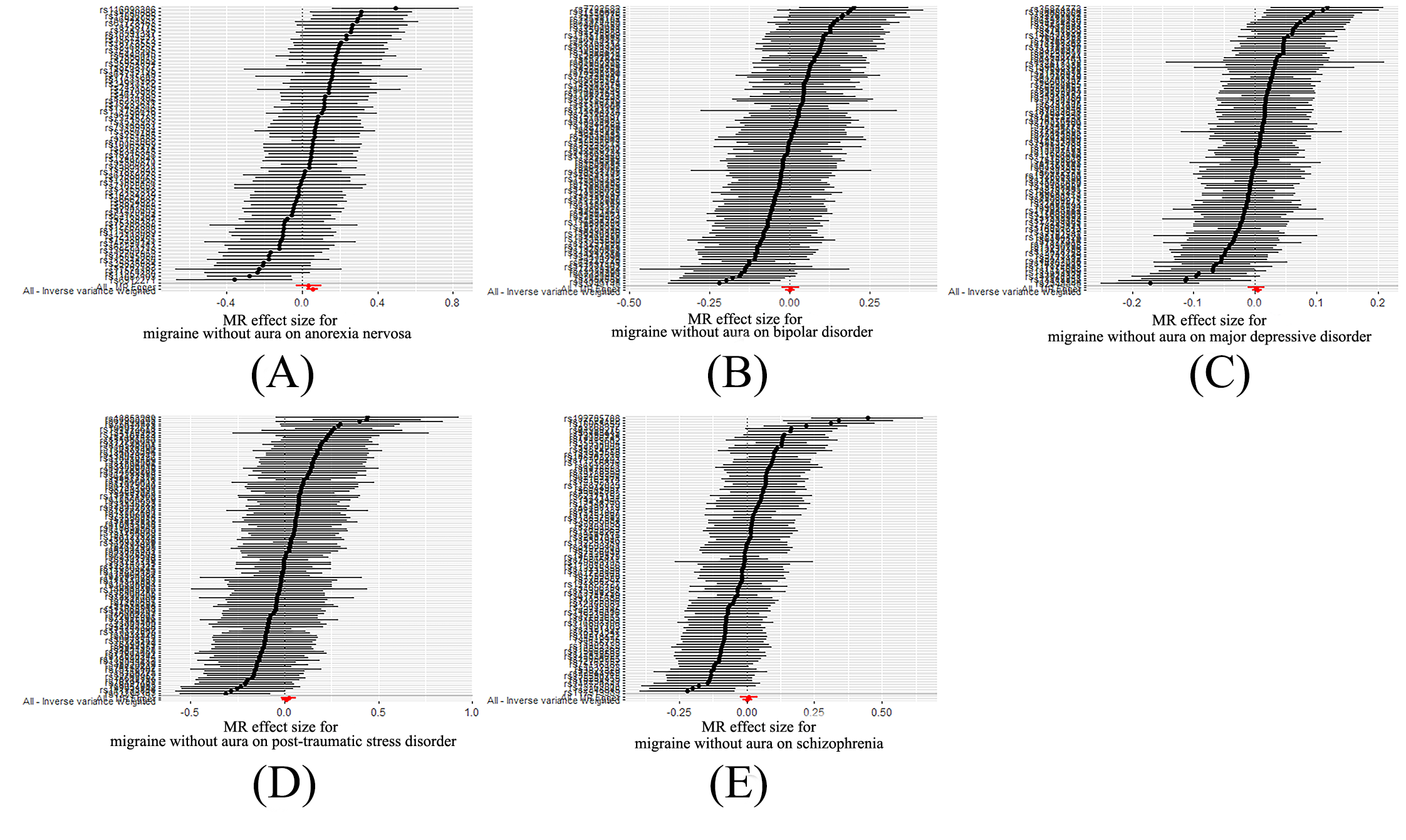

Supplement: SUPPLEMENTARY FIGURE S8 — Forest plot of the causal effect of migraine without aura on psychiatric disorders and associated SNPs. [file Image_8.TIF]
